# Identification of candidate regulatory genes for intramuscular fatty acid composition in pigs by transcriptome analysis

**DOI:** 10.1186/s12711-024-00882-x

**Published:** 2024-02-12

**Authors:** Jesús Valdés-Hernández, Josep M. Folch, Daniel Crespo-Piazuelo, Magí Passols, Cristina Sebastià, Lourdes Criado-Mesas, Anna Castelló, Armand Sánchez, Yuliaxis Ramayo-Caldas

**Affiliations:** 1 Plant and Animal Genomics, Centre for Research in Agricultural Genomics (CRAG), CSIC-IRTA-UAB-UB, Campus UAB, Bellaterra, Spain; 2 Departament de Ciència Animal i dels Aliments, Facultat de Veterinària, Universitat Autònoma de Barcelona, Bellaterra, Spain; 3 Departament de Genètica i Millora Animal, Institut de Recerca y Tecnologia Agraroalimentàries (IRTA), Caldes de Montbui, Spain

## Abstract

**Background:**

Intramuscular fat (IMF) content and its fatty acid (FA) composition are typically controlled by several genes, each with a small effect. In the current study, to pinpoint candidate genes and putative regulators involved in FA composition, we performed a multivariate integrative analysis between intramuscular FA and transcriptome profiles of porcine *longissimus dorsi* (LD) muscle. We also carried out a combination of network, regulatory impact factor (RIF), in silico prediction of putative target genes, and functional analyses to better support the biological relevance of our findings.

**Results:**

For this purpose, we used LD RNA-Seq and intramuscular FA composition profiles of 129 Iberian × Duroc backcrossed pigs. We identified 378 correlated variables (13 FA and 365 genes), including six FA (C20:4*n*-6, C18:2*n*-6, C20:3*n*-6, C18:1*n*-9, C18:0, and C16:1*n*-7) that were among the most interconnected variables in the predicted network. The detected FA-correlated genes include genes involved in lipid and/or carbohydrate metabolism or in regulation of IMF deposition (e.g., *ADIPOQ*, *CHUK*, *CYCS*, *CYP4B1*, *DLD*, *ELOVL6*, *FBP1*, *G0S2*, *GCLC*, *HMGCR*, *IDH3A*, *LEP*, *LGALS12*, *LPIN1*, *PLIN1*, *PNPLA8*, *PPP1R1B*, *SDR16C5*, *SFRP5*, *SOD3*, *SNW1*, and *TFRC*), meat quality (*GALNT15*, *GOT1*, *MDH1*, *NEU3*, *PDHA1*, *SDHD*, and *UNC93A*), and transport (e.g., *EXOC7* and *SLC44A2*). Functional analysis highlighted 54 over-represented gene ontology terms, including well-known biological processes and pathways that regulate lipid and carbohydrate metabolism. RIF analysis suggested a pivotal role for six transcription factors (CARHSP1, LBX1, MAFA, PAX7, SIX5, and TADA2A) as putative regulators of gene expression and intramuscular FA composition. Based on in silico prediction, we identified putative target genes for these six regulators. Among these, *TADA2A* and *CARHSP1* had extreme RIF scores and present novel regulators in pigs. In addition, the expression of *TADA2A* correlated (either positively or negatively) with C20:4*n*-6, C18:2*n*-6, C20:3*n*-6, C18:1*n*-9, and that of *CARHSP1* correlated (positively) with the C16:1*n*-7 lipokine. We also found that these two transcription factors share target genes that are involved in lipid metabolism (e.g., *GOT1*, *PLIN1*, and *TFRC*).

**Conclusions:**

This integrative analysis of muscle transcriptome and intramuscular FA profile revealed valuable information about key candidate genes and potential regulators for FA and lipid metabolism in pigs, among which some transcription factors are proposed to control gene expression and modulate FA composition differences.

**Supplementary Information:**

The online version contains supplementary material available at 10.1186/s12711-024-00882-x.

## Background

Fatty acids (FA) are crucial for living organisms, as they serve as important energy sources and, in humans, they are known to play an important role in health. Furthermore, FA composition plays a significant role in meat quality in pigs [1], including technological and sensorial quality of meat products [2]. Fatty acids can be classified as saturated (SFA, absence of double bonds) or unsaturated (presence of one or more double bonds) with monounsaturated (MUFA) and polyunsaturated (PUFA).

Fat, liver, and muscle are important tissues for FA metabolism and have different FA compositions, which are affected by several factors including genetic, management and environmental factors, and by gene expression, among others. Notwithstanding, the relationship between FA composition and gene expression is complex and still not fully elucidated. For instance, the compositional distribution of lipids in pig muscle correlates with intramuscular fat content (IMF, also referred to as marbling). Specifically, the deposition of FA classes when neutral lipid content is adjusted for IMF fractionation as a covariate suggests that deposition of SFA and MUFA increases significantly with age from 6 to 9 months, whereas that of PUFA decreases significantly [3]. For complex phenotypes such as porcine IMF content and its FA composition, in a previous study based on a hierarchical clustering analysis [4], we have shown that lipogenic-related genes are in general positively correlated with MUFA, while the lipolytic-related genes are specifically positively correlated with PUFA. Likewise, by conducting RNA sequencing (RNA-Seq) experiments, several studies have reported numerous candidate genes with differential effects or global changes on intramuscular FA composition across several tissues, such as backfat, liver, and muscle [5–9]. Our research group has also identified links between the muscle transcriptome and its FA profile in Iberian × Duroc backcross (BC1_DU) pigs [9]. In that study, we used a univariate extended linear model and found that several candidate genes involved in carbohydrate and lipid metabolism were significantly associated with FA composition traits (mainly MUFA, PUFA, and FA ratios). Other relevant associations with FA ratios (e.g., ω6/ω3 and C18:2*n*-6/C18:3*n*-3, both including major FA) and their main transcriptional regulators still remain to be investigated. A better understanding of biological mechanisms that underlie complex traits requires integrative approaches via gene networks [10], as well as the identification of the main regulators driving gene-by-gene interactions [11]. In the context of such multivariate and integrative approaches, there are several tools that allow the exploration and integration of biological datasets with a focus on variable selection. Among these, the mixOmics R package includes a plethora of multivariate methodologies with extensive statistical approximations [12].

In the study reported here, we analyzed our existing muscle data (i.e., expression levels of genes and only individual FA phenotypes) of the BC1_DU population [9]. From a multivariate perspective and using a complementary integrative analysis, we investigated the gene expression of potential candidate genes and novel regulators for intramuscular FA composition. We focused on integrative analyses between intramuscular FA and gene expression profiles in pigs to identify representative FA phenotypes, key regulators, candidate genes, and biological processes and metabolic pathways related to the FA composition in muscle.

## Methods

### Animals and phenotypic data

The experimental backcross population (25% Iberian and 75% Duroc, BC1_DU) used in this study is described in [9]. Briefly, it included 129 animals, which were raised under the standard, intensive conditions of production; and fed ad libitum with a cereal-based commercial diet and free access to water. Details on the experimental BC1_DU generation, animal raising, and feeding are in [13]. Animal procedures were carried out according to the Spanish Policy for Animal Protection RD1201/05, which meets the European Union Directive 86/609 about the protection of animals used in experimentation. This study was conducted in accordance with relevant guidelines and regulations of the animal care and use committee of the *Institut de Recerca i Tecnologia Agroalimentàries* (IRTA), which adopts “The European Code of Conduct for Research Integrity”. The experimental protocol was approved by the Ethical Committee of the IRTA. Our study is also reported in full compliance with the ARRIVE guidelines (https://arriveguidelines.org/).

Animals were slaughtered in the same commercial abattoir in Mollerussa (Spain). Samples of the *longissimus dorsi* (LD) skeletal muscle (59 females and 70 non-castrated males) distributed in five slaughterhouse batches were collected, immediately snap frozen in liquid nitrogen and stored at − 80 °C until analysis. At slaughter, the average age of the pigs was 190 days (ranging from 174 to 205 days), with an average carcass weight (CW) of 73.70 kg (ranging from 46.10 to 109.20 kg).

The composition of FA in the chain-length range of C14-C20 in LD muscle (n = 129) was determined using a gas chromatography of methyl esters protocol [14]. Briefly, 200 g of muscle sample from each BC1_DU pig were homogenized and used to measure the FA profile in duplicate. Crespo-Piazuelo et al. [15] provide additional information on the muscle FA composition in the BC1_DU population. Then, each individual FA methyl ester (n = 15 FA phenotypes) was quantified and expressed as a percentage of the total amount of FA (Table 1).

**Table 1:** Descriptive statistics of the FA composition phenotypes measured in percentages in the *longissimus dorsi* muscle from BC1_DU pigs

Trait	Name	Mean	SD	Min	Max	SE	CV^a^
C14:0	Myristic acid	1.27	0.23	0.73	1.78	0.02	17.95
C16:0	Palmitic acid	23.91	1.65	18.69	27.59	0.15	6.90
C18:0	Stearic acid	14.38	1.69	8.81	19.90	0.15	11.78
C20:0	Arachidic acid	0.23	0.08	0.08	0.71	0.01	33.94
C16:1*n*-7	Palmitoleic acid	2.79	0.53	1.24	4.08	0.05	18.83
C16:1*n*-10	Sapienic acid	0.30	0.12	0.16	0.92	0.01	38.62
C18:1*n*-9	Oleic acid	35.93	5.71	19.99	44.15	0.50	15.88
C18:1*n*-7	Vaccenic acid	3.82	0.30	3.02	4.83	0.03	7.97
C20:1*n*-9	Gondoic acid	0.73	0.16	0.35	1.48	0.01	22.38
C18:2*n*-6	Linoleic acid	12.13	5.82	4.81	29.34	0.51	48.01
C18:3*n*-3	α-Linolenic acid	0.40	0.13	0.15	0.89	0.01	32.77
C20:2*n*-6	Eicosadienoic acid	0.43	0.12	0.14	0.91	0.01	28.61
C20:3*n*-3	Eicosatrienoic acid	0.18	0.10	0.02	0.65	0.01	54.98
C20:3*n*-6	Dihomo-gamma-linolenic acid	0.45	0.29	0.09	1.49	0.03	63.29
C20:4*n*-6	Arachidonic acid	2.58	1.94	0.47	10.51	0.17	75.01

^a^Coefficient of variation (%)

### Gene expression data

Total RNA was isolated from a muscle sample (100 mg) of each of the 129 animals using the RiboPure™ Isolation kit for High-Quality Total RNA (Ambion^®^; Austin, TX, USA) following the manufacturer’s recommendations. RNA quantification and purity were estimated with a NanoDrop ND-1000 spectrophotometer (NanoDrop products, Wilmington, DE, USA). RNA integrity was checked by an Agilent Bioanalyzer-2100 (Agilent Technologies, Inc., Santa Clara, CA, USA), and samples with an RNA integrity number (RIN) greater than 7 were used for the RNA-Seq experiment.

Library preparation and sequencing were performed at the CNAG institute (*Centro Nacional de Análisis Genómico*, Barcelona, Spain). For each sample, one paired-end library with an insert size of approximately 300 bp was prepared using the TruSeq Stranded mRNA kit (Illumina, Inc.; San Diego, USA). Libraries were labeled by barcoding, pooled, and run on the Illumina HiSeq 3000/HiSeq4000 systems (Illumina, Inc.; San Diego, USA), yielding on average 45.09 million 2 × 75 bp paired-end reads per sample.

### Bioinformatic and statistical analyses

Quality control and basic statistics of the sequencing data were performed using the FastQC (v0.11.9) [16] and MultiQC v0.7 [17] programs. Sequencing reads were mapped with the STAR software (v2.7.9a) using default parameters [18], and the *Sscrofa11.1* pig genome assembly as reference. On average, 90.1% of reads were uniquely mapped (ranging from 80.5 to 96.1%). Gene expression was quantified by the RSEM (v1.2.28) software [19] using default parameters and annotation from Ensembl Pig Genes 97.

Pre-processing of the raw count matrix was performed by filtering based on a minimum of 129 reads per row and 15,091 genes were retained for further analyses. The raw count data were transformed into counts per million (CPM) to normalize the values (i.e., with log = TRUE and prior.count = 1 arguments) using the edgeR v3.38.1 package [20]. Then, the 15,091 retained genes were matched against the newer annotation from Ensembl Pig Genes 104 (*Sscrofa11.1*) using the biomaRt package [21] v2.52.0, which left 12,381 genes with a gene name or symbol.

A regularized canonical correlation analysis (rCCA) was performed using the expression dataset of the 12,381 genes (matrix **Y** ) and the 15 FA traits (matrix **X**) measured on the 129 individuals. The rCCA multivariate approach is implemented in the mixOmics v6.14.1 package [12], which allows subsets of canonical variables that maximize the correlation between two datasets (**X** and **Y**, respectively of sizes n × p and n × q) to be identified [22]. The shrinkage method was used to tune out the regularization parameters (λ1 and λ2) with values of λ1 = 0.05 and λ2 = 0.15, and ncomp = 3. Rather than considering all the genes that were included in the first canonical component (CC1) and according to the previous estimate by Ramayo-Caldas et al. [23], we applied a conservative approach and only kept genes for which the correlation between gene expression and FA traits was at least 0.29.

To display the rCCA results and improve their interpretation, we applied three graphical outputs (circle plot, network, and Clustered Image Maps) that are all implemented in the mixOmics package [12] via *plotVar*, *network* and *cim* functions. In particular, the generated network output was exported to Cytoscape file format using the igraph v1.3.2 package [24]. In the network [25], FA were filled with colors to facilitate the identification of each group (i.e., SFA, MUFA and PUFA) and the connected genes. Likewise, the genes were filled with different colors according to their functional group or gene family using a pre-built list according to their functions (see the ‘Gene functional information’ section). In addition, we used a complementary heatmap via the ComplexHeatmap v2.14.0 package [26] to illustrate the different clusters of variables and the degree of correlation between them.

For the functional analysis, genes included in the CC1 (cutoff of r ±|0.29|) were submitted to the ClueGO v2.5.4 plugin [27] in the Cytoscape v3.7.1 software [25], using default parameters. Gene ontology (GO) significance was assessed with a hypergeometric test, keeping only the GO terms (biological processes, molecular function, and pathways) that had a corrected Benjamini–Hochberg (BH) *P*-value lower than 0.05 [28]. All genes expressed in muscle (12,381) were included as background in this step. Furthermore, GO tree interval levels were set from three to eight and a minimum k-score of 0.44 and a minimum of three genes per cluster with at least 4% in selected genes were used. Results with and without the fusion feature “GO Term Fusion” were generated to evaluate the redundant parent–child terms. In addition, we visualized the ClueGO output using an R script via the ggplot2 v3.3.5 package [29], which allowed us to identify the intersection of significantly associated genes according to over-represented GO terms.

### Analysis of regulatory impact factors (RIF)

The RIF analysis was conducted using the *runAnalysis* function of the CeTF v1.8.0 package [30]. The RIF algorithm is described in detail in Reverter et al. [11]. Briefly, the RIF metrics (RIF1 and RIF2) aim at identifying relevant regulators (i.e., transcription factors, TF) from the gene expression data. This step calculates, for each condition, the co-expression correlation between the TF and the differentially expressed (DE) genes. For the DE analysis, we created two conditions by classifying samples according to their FA profile through a principal component analysis (PCA). In the PCA, the *prcomp* function with scale = TRUE was used, considering as input the composition data of the pre-selected FA from the rCCA. In fact, we chose the extreme values from PC1 (condition 1 and condition 2 with n = 60, i.e., 30 samples per condition). Here, animals in condition 1 included 10 females and 20 males while those in condition 2 included 15 females and 15 males, in both cases belonging to five slaughterhouse batches. The *fviz_pca* function of the factoextra v1.0.7 package was used to extract and visualize the PCA results [31], including the FA profile according to the three classes (SFA, MUFA and PUFA). Significant differences (corrected *P*-value < 0.05) between the means of FA conditions and phenotypes, for each FA selected in the rCCA were determined using a t-test approach, and the standard error of the mean (SEM) was calculated. We also tested the correlation between the FA used in the selection of the two groups for DE gene analysis and we identified the TF present in the expression data based on the list of pig TF available in the AnimalTFDB v3.0 database (http://bioinfo.life.hust.edu.cn/AnimalTFDB/#!/).

### Analysis of the target genes of the transcription factors

The list of TF used in the RIF step was also examined to identify in silico putative target genes with an expression that was DE between the two FA conditions (n = 30 samples per condition). This complementary analysis was conducted using the *SmearPlot* function [30]. Briefly, the predicted target genes identified by this approach were extracted, and, for each condition, co-expression between the TF and the DE genes was calculated using the partial correlation with information theory (PCIT) algorithm. The resulting matrix contained the correlations between genes, and a separate object included information about the significant correlations (adjusted values, padj = 0.05) of target genes (lfc = 1.5 and padj = 0.05) and the TF of interest.

### Functional classification of FA-interconnected genes in the network

To facilitate functional classification of the candidate genes, we ranked the global list of rCCA-derived genes. Overall, we focused on the functional annotation of genes and their plausible function in different aspects of FA, lipid, and carbohydrate metabolisms (see Additional file 1: Tables S1, S2 and S3). First, we used information from the ClueGO analysis that divided the genes into different functional groups, which contained biological processes and pathways clustered according to GO term similarities. Second, a trained list was created using the GUILDify v2.0 tool [32], which included genes associated with predefined keywords such as: “adipokine”, "amino acid metabolism”, “electron transport chain”, “enzyme”, “fatty acid beta-oxidation”, “fatty acid metabolism”, “fatty acid synthesis”, “gluconeogenesis”, “glucose metabolism”, “glycolysis”, “tricarboxylic acid cycle”, “lipid metabolism”, “carbohydrate metabolism”, “lipid degradation”, “lipid synthesis”, “nucleic acid”, “nucleotide metabolism”, “nutrient”, “receptor”, “transporter”, and “energy homeostasis”; as well as *homo sapiens* species and lipogenic tissues (adipose, liver and muscle-skeletal) options. Briefly, GUILDify uses the biological interaction and network analysis (BIANA) knowledge database to create a species-specific interaction network for each gene detected. In the current study, the *netcombo* prioritization algorithm based on network topology, and the highest guild score for the top 100 gene products (with only seed) were considered to constitute such a list. Third, the presence of TF and cofactors in pigs was corroborated according to the annotation of the aforementioned AnimalTFDB v3.0 database. Thus, the gene functional classification was based on the potential biological functions that were compiled from the overlap of the rCCA-derived gene list with the three previously explained information sources.

## Results

### Regularized canonical correlation analysis (rCCA)

**Fig. 1:**
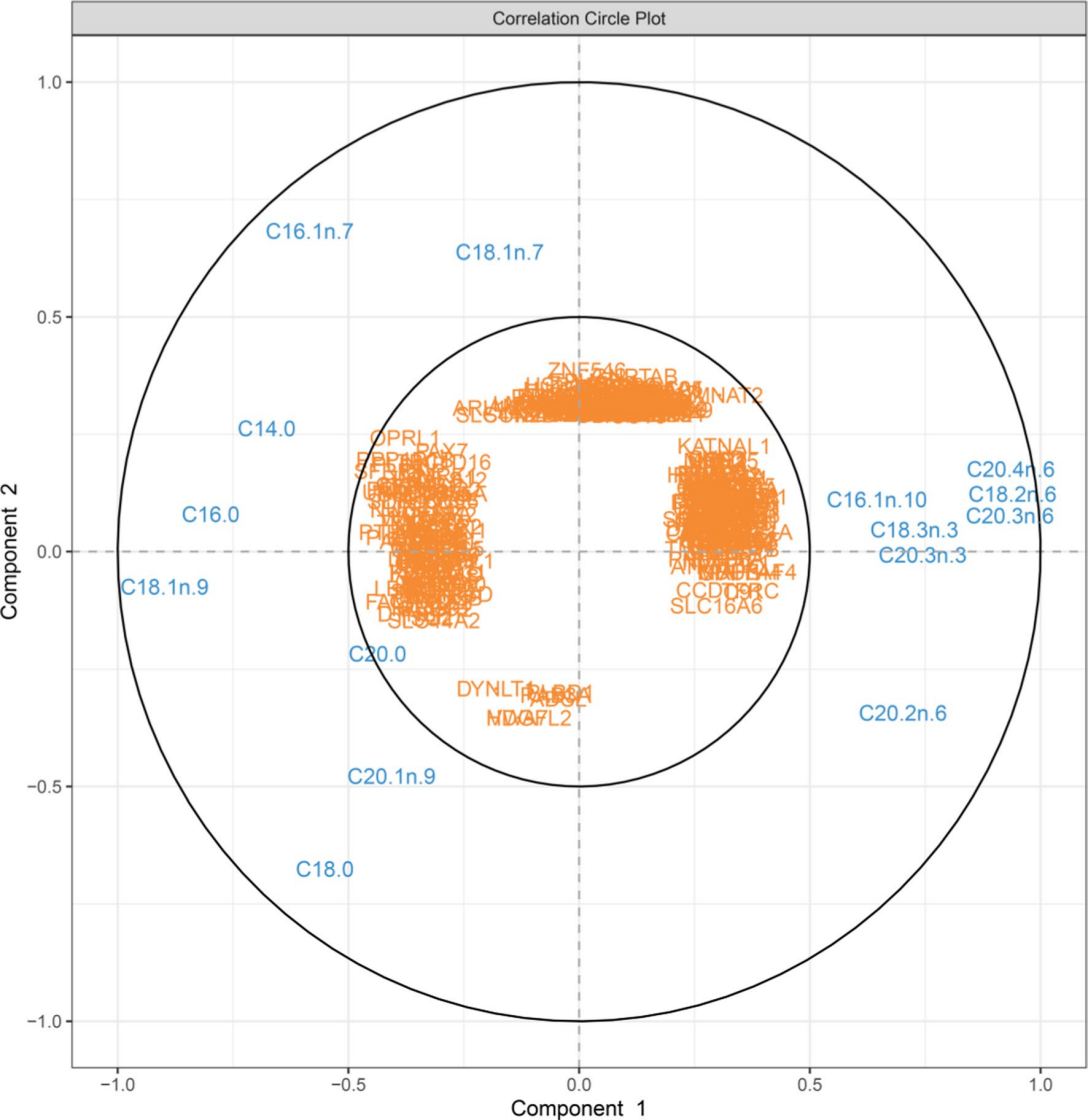
Correlation circle plot from the PCA applied to the FA phenotypes and gene expression in muscle of BC1_DU pigs for the first two rCCA dimensions (15 FA and 365 genes selected in total)

Here, we used a multivariate integrative approach to explore the relationship between the muscle transcriptome (n = 12,381 genes) and intramuscular FA composition (n = 15 FA) of 129 BC1_DU pigs. The correlation structure between datasets of interest is shown in Fig. 1. The rCCA yielded 365 genes and 15 FA (see Additional file 2: Table S4) that were included in the first canonical component (CC1). As expected, our results revealed that CC1 separated both SFA (C16:0) and MUFA (C18:1*n*-9) from PUFA (C18:3*n*-3, C18:2*n*-6, C20:2*n*-6, C20:3*n*-3, C20:3*n*-6 and C20:4*n*-6), while the second canonical component (CC2) differentiated C16:1*n*-7 and C18:1*n*-7 MUFA from C18:0 SFA. An additional correlation circle plot from the PCA with contribution variables according to CC1 versus the third canonical component (CC3) is presented in Additional file 3: Fig. S1.

The most relevant variables participating in the definition of each component were C16:1*n*-7, C18:1*n*-9 and C18:0, as well as C18:2*n*-6, C20:3*n*-6, C20:4*n*-6, C20:3*n*-3 and C20:2*n*-6 (Fig. 1). For example, we observed a relevant contribution of C18:2*n*-6, C20:3*n*-6, C20:4*n*-6 (positive) and C18:1*n*-9, C16:0 (negative) on component 1, and C16:1*n*-7 (positive) and C18:0 (negative) on component 2. Likewise, Fig. 1 suggests a negative correlation between [C18:2*n*-6, C20:3*n*-6, C20:4*n*-6, C18:3*n*-3] and C18:1*n*-9. Notably, the 365 genes were clustered in four groups at radii ~ 0.29 (with prioritized variables of gene expression) (Fig. 1). Details on the genes correlated with each FA (with and without the cutoff) are in Additional file 2: Table S4.

**Fig. 2:**
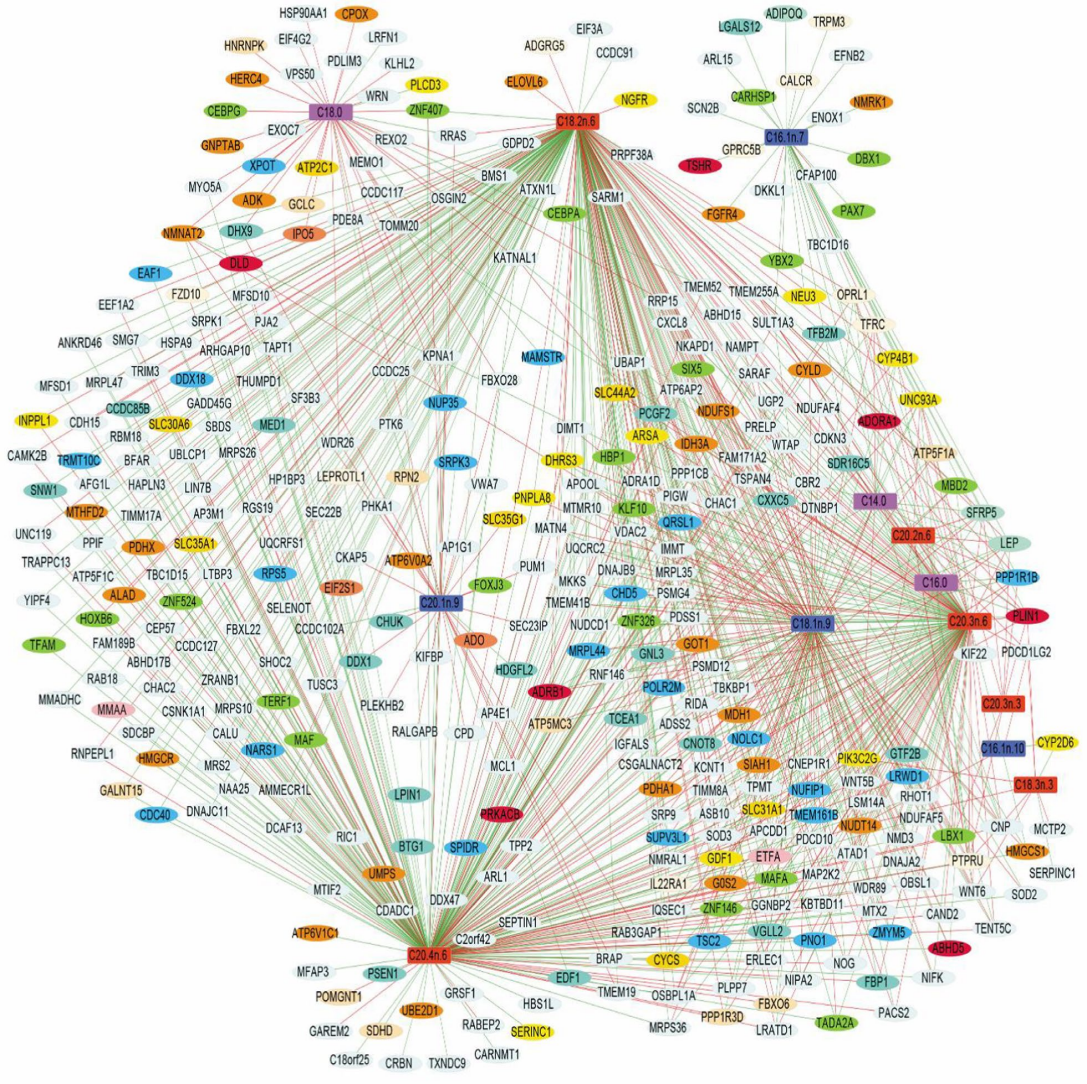
Network plot for the *longissimus dorsi* muscle study in BC1_DU pigs. Green and red edges indicate positive and negative correlations. Output obtained for the first three rCCA dimensions (13 FA and 365 genes were selected), showing the correlation structure for all bipartite relationships with a correlation cutoff of 0.29. Color legend: FA: magenta = SFA members; royal blue = MUFA members; orange red = PUFA members; and genes: dark orange = enzyme; aquamarine = adipokine; chartreuse = TF; turquoise = TF cofactors; yellow = lipid metabolism‐related genes; Navajo white = carbohydrate metabolism; crimson = glycolysis; gold = transporter; light pink = fatty acid beta-oxidation; coral = amino acid metabolism; corn silk = receptor family; deep sky blue = nucleic acid metabolism. Out of a total of 365 genes, the 176 colored genes refer to a functional or gene family classification (while 189 genes were unclassified)

The results of the network approach suggested a complex correlation structure (bipartite relationship) between the FA profile and gene expression data (Fig. 2). Simultaneously, the representation of correlations via edges indicated that positive relationships were more common than negative relationships (i.e., lines in green and in red, respectively, in Fig. 2). As shown in Fig. 2, the network added another layer of information, which allowed visualization of groups of variables (FA and genes). Our results show that 13 FA and 365 genes (378 nodes in total) were selected. Among the 13 FA, five (C16:1*n*-7, C20:1*n*-9, C18:0, C18:2*n*-6, and C20:4*n*-6) were correlated with a list of specific genes for each FA (see Additional file 2: Table S4). The fourth most interconnected FA (see Additional file 2: Table S4) were C20:4*n*-6, C18:2*n*-6, C20:3*n*-6 and C18.1*n*-9 (Fig. 2). Remarkably, both C20:4*n*-6 and C18:2*n*-6 showed the largest number of correlated genes, with 87% of the genes in common. In particular, C18:1*n*-9 was grouped very close to seven other FA (C14:0, C20:3*n*-3, C20:2*n*-6, C20:3*n*-6, C16:1*n*-10, C18:3*n*-3 and C16:0) (Fig. 2). In fact, C20:3*n*-6 and C18:1*n*-9 were the second pair of FA with the largest number of correlated genes, with 80.5% of genes in common. Please note, that the subsets of connected genes suggested more complex relationships with the presence of shared or specific genes for FA.

It is worth mentioning that we observed a set of 10 rCCA-derived genes (*PLIN1*, *UNC93A*, *SFRP5*, *PPP1R1B*, *LEP*, *OPRL1*, *PTPRU*, *NUDT14*, *TFRC,* and *CYP4B1*) that showed the largest number of connections with FA (i.e., 7 to 10 connections out of 13 FA). In addition, six other rCCA-derived genes (*CNP*, *LBX1*, *NDUFAF4*, *NMNAT2*, *PIK3C2G*, and *SDR16C5*) showed at least six connections with total FA (Fig. 2) (for details [see Additional file 2: Table S4]). The *LBX1*, *PIK3C2G*, and *SDR16C5* genes were positively correlated with C16:0 and C18:1*n*-9 and negatively correlated with C18:2*n*-6, C20:3*n*-6 and C20:4*n*-6, and only the *PIK3C2G* gene was negatively correlated with C18:3*n*-3. However, we also detected 107 genes that were uniquely correlated with a specific FA (Fig. 2) and (see Additional file 2: Table S4). For instance, the *CEBPG* and *GCLC* genes were negatively correlated with C18.0, while *EXOC7* was positively correlated with C18.0. Genes such as *ADIPOQ*, *LGALS12*, *PAX7*, *CARHSP1* and *YBX2* were positively correlated with C16:1*n*-7. As other examples, the *CEBPA*, *ELOVL6*, and *MCTP2* genes were correlated negatively with C18:2*n*-6 and C18:3*n*-3, respectively; while *SDHD*, *LPIN1*, *DLD*, and *SNW1* were positively correlated with C20:4*n*-6, and *HMGCR*, *LEPROTL1*, and *TCEA1* were positively correlated with C18:2*n*-6 and C20:4*n*-6. The *PNPLA8*, *HMGCS1*, *KATNAL1*, and *ATAD1* genes were negatively correlated with C18:1*n*-9, but positively correlated with C18:2*n*-6 and C20:4*n*-6 (Fig. 2).

A complementary heatmap illustrated the hierarchical clustering of the variables (13 FA and 365 genes) and the degree of correlation between them (see Additional file 4: Fig. S2). In general, two large clusters of FA and four clusters of genes were observed. Regarding the FA, cluster 1 was composed of six FA (C16:1*n*-7, C18:0, C20:1*n*-9, C18:1*n*-9, C16:0 and C14:0), while cluster 2 grouped the other seven FA (C20:2*n*-6, C16:1*n*-10, C18:3*n*-3, C20:3*n*-3, C20:3*n*-6, C20:4*n*-6, and C18:2*n*-6). For the four gene clusters, a variable number of genes was detected in each of them. Notably, cluster 1 grouped together 41 genes, including the nine genes showing the greatest interconnection with FA, five TF (*CARHSP1*, *CEBPA*, *DBX1*, *PAX7*, and *YBX2*), plus 27 protein-coding genes including those functionally related to lipid metabolism (e.g., *ELOVL6*, *FGFR4*, *LGALS12*, *NEU3*, *NGFR*, *NMRK1*, *NUDT14*, *PIK3C2G*, *SDR16C5*, and *WNT6*). The two major gene clusters were 4 and 2, in which we detected several genes involved in energy and lipid metabolism [e.g., cluster 2: *G0S2*, *SOD3*, *SLC44A2*, *PLCD3*, *EDF1*, *GDF1*, and *FBP1*; cluster 4: *CYCS*, *CYLD*, *EIF3A*, *GALNT15*, *GOT1*, *HMGCR*, *IDH3A*, *LPIN1*, *MDH1*, *MRPL44*, *MTIF2*, *NAMPT*, *NMNAT2*, *PDHA1*, *PNPLA8*, *SLC31A1*, *TFRC*, and *UGP2*] and other TF [e.g., cluster 2: *LBX1, MAFA*, and *SIX5*; cluster 4: *CEBPG*, *HBP1*, *KLF10*, *TADA2A*, *TERF1*, and *TFAM*]. Finally, cluster 3 contained the smallest number of genes, most of which are not well described in the literature. As an example, the *CHUK* (encoding a component of a cytokine-activated protein complex)*, HSP90AA1,* and *GCLC* genes are part of cluster 3 and are related to lipid or glucose metabolism. Details on the genes contained in each cluster and their respective correlation with FA are in Additional file 4: Fig. S2 and Additional file 2: Table S4, respectively. In addition, details on the distribution of correlations of FA with gene expression based on a density heatmap (with quantiles and mean values) are in Additional file 5: Fig. S3.

### Functional analysis of genes correlated with FA

**Fig. 3:**
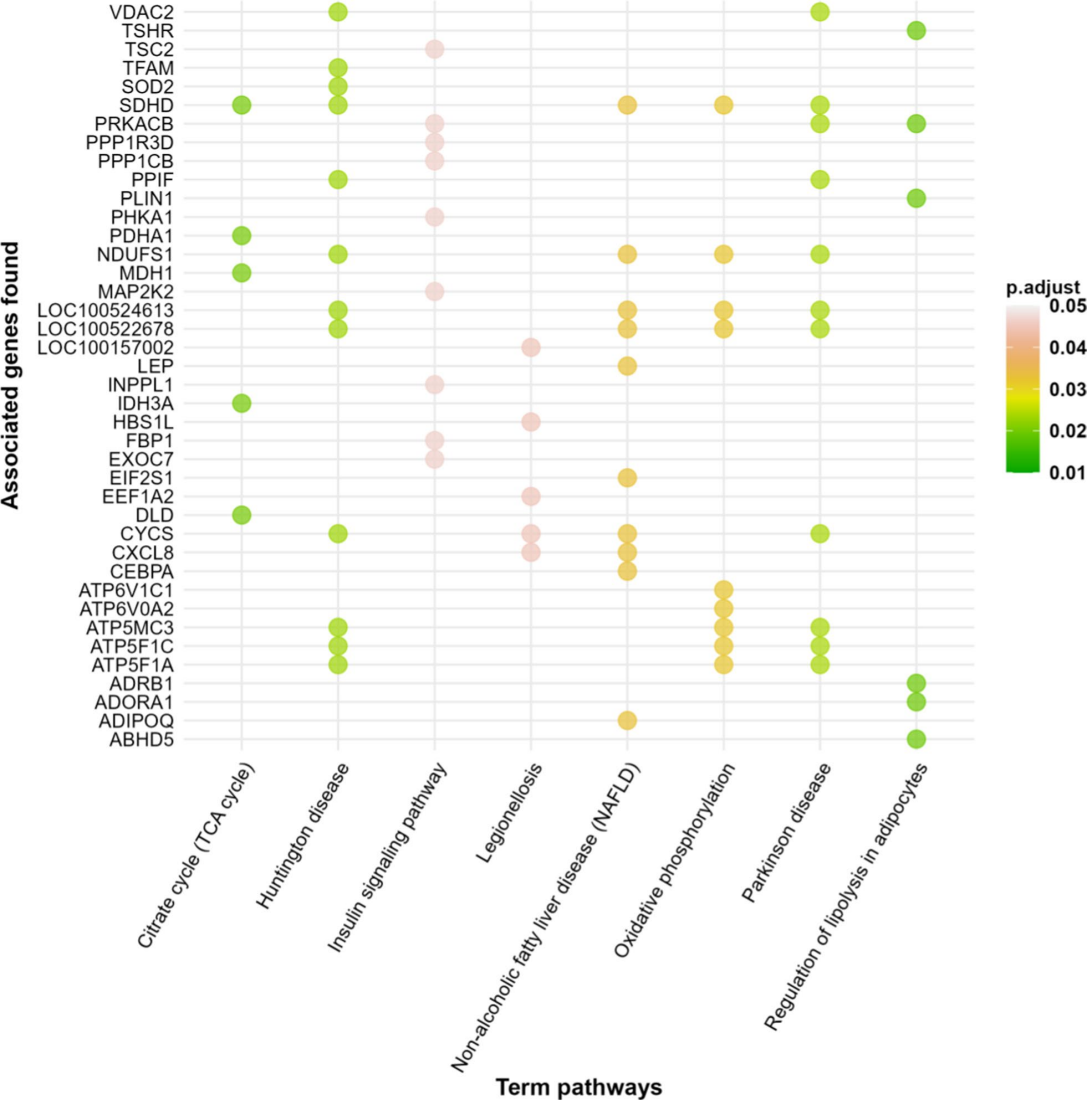
Functional analysis of the correlated genes from the rCCA approach that were significantly enriched in GO terms according to metabolic pathway delimitation. This output is a representation of the original table of generated with the ClueGO plugin in the Cytoscape software

The 365 genes selected by rCCA were submitted to a GO analysis. Fifty-four GO terms (8 molecular functions, 8 pathways, and 38 biological processes) (see Additional file 7: Table S5) were significantly over-represented (BH corrected *P*-value < 0.05). In total, 125 genes were annotated into different functional groups, including an enrichment in GO terms related to lipid and carbohydrate metabolism. Notably, some of the closely associated Kyoto encyclopedia of genes and genomes (KEGG) pathways were “regulation of lipolysis in adipocytes”, “citrate cycle (TCA cycle)”, “non-alcoholic fatty liver disease (NAFLD)”, “oxidative phosphorylation” and “Insulin signaling pathway” (Fig. 3).

The GO analysis also suggested that these genes were significantly enriched in biological processes (see Additional file 6: Fig. S4 with “GO Term Fusion”), such as “mitochondrial gene expression”, “tricarboxylic acid cycle”, “electron transport chain”, “ATP hydrolysis coupled cation transmembrane transport”, “regulation of response to nutrient levels”, “magnesium ion transmembrane transport”, “generation of precursor metabolites and energy”, and “respiratory electron transport chain”. The complete results of GO terms with and without “GO Term Fusion” are listed in Additional file 7: Table S5.

### Analysis of regulatory impact factors (RIF)

**Fig. 4:**
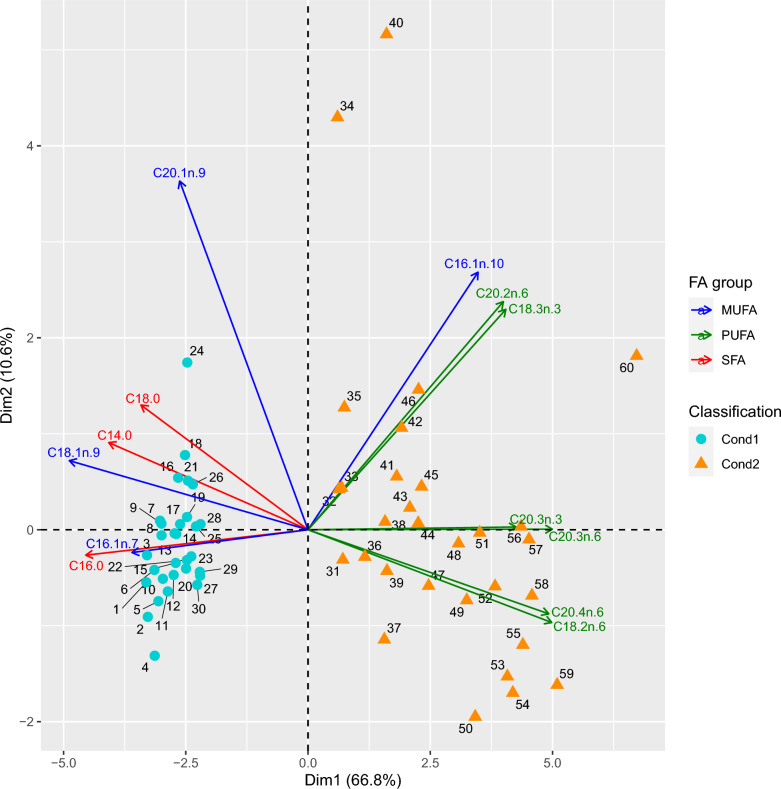
Principal component analysis summarizing the separation and similarities among FA profiles for the pigs with extreme FA values. PC1 and PC2 explained 77.40% of the total variance. The visualization of the eigenvectors was implemented to delimit the FA belonging to their respective group (SFA, MUFA, PUFA)

Based on the output of the rCCA, 22 putative regulators (i.e., TF) were identified. To perform the RIF analysis, the dataset was split into two conditions (30 individuals in each) based on the FA profile from the PCA data (Fig. 4). Condition 1 included animals that had a FA profile with fewer SFA and MUFA and more PUFA (and a lower IMF content) and condition 2 was the opposite with more SFA and MUFA and fewer PUFA, concurrently with a higher IMF content (see Additional file 8: Table S6).

We also found a complex correlation pattern between components of the three classes of FA (SFA, MUFA and PUFA) in muscle. For example, some FA, such as C18:0 SFA and C18:1*n*-9 MUFA were positively correlated with each other but negatively with the most abundant PUFA (C18:2*n*-6, C18:3*n*-3, and C20:4*n*-6), which were positively correlated with each other. Correlations between the FA are in Additional file 9: Table S7. The results of the DE analysis revealed 293 DE genes (i.e., 274 genes and 19 TF) (lfc = 1.5 and padj = 0.05) between the two FA conditions (see Additional file 10: Table S8).

Table 2 contains a summary of the 19 regulators that were identified by including the RIF parameters. To interpret these results, it is important to note that information from RIF1 and RIF2 is complementary. The RIF1 score classified the TF that were the most differentially co-expressed with the highly abundant and highly DE genes, whereas the RIF2 score classified the TF that had the best ability to act as potential predictors of the abundance of DE genes due to FA differences. Among them, the top five TF with extreme values for the RIF metrics (Table 2) are the following, with a positive score: *CARHSP1*, *TERF1*, *CEBPG*, *TFAM*, *MAF*; and with a negative score: *TADA2A*, *MBD2*, *HBP1*, *SIX5*, and *FOXJ3*); and for RIF2 with a positive score: *KLF10*, *TADA2A*, *HBP1*, *FOXJ3*, and *ZNF407*, and with a negative score *MBD2*, *SIX5*, *TERF1*, *CEBPG*, and *MAF*. Based on the absolute values of RIF, we found that the first and second most relevant regulators were TADA2A and CARHSP1 based on RIF1 and KLF10 and TADA2A based on RIF2 (Table 2). Table 2 also shows that the TF were classified into ten different families (i.e., based on AnimalTFDB3.0 database).

**Table 2:** RIF analysis with 19 significant TF^a^ identified by RIF1 or RIF2 metrics according to extreme FA profiles and gene expression in BC1_DU pigs

TF	Gene description	Family	Avgexpr^b^	RIF1	RIF2
*TADA2A*	Transcriptional adaptor 2A	MYB	4.11	− 2.09	1.44
*MBD2*	Methyl-CpG binding domain protein 2	MBD	4.90	− 1.43	− 1.44
*SIX5*	SIX homeobox 5	Homeobox	2.38	− 0.83	− 1.30
*TERF1*	Telomeric repeat binding factor 1	MYB	3.21	1.63	− 1.28
*CEBPG*	CCAAT enhancer binding protein gamma	TF_bZIP	4.96	1.49	− 1.15
*HBP1*	HMG-box transcription factor 1	HMG	6.12	− 0.89	1.12
*FOXJ3*	Forkhead box J3	Fork_head	6.36	− 0.54	1.09
*CARHSP1*	Calcium regulated heat stable protein 1	CSD	3.37	1.86	0.82
*MAF*	MAF bZIP transcription factor	TF_bZIP	7.19	0.39	− 0.68
*TFAM*	Transcription factor A, mitochondrial	HMG	4.79	0.82	− 0.52
*KLF10*	Kruppel like factor 10	zf-C2H2	6.24	− 0.32	1.53
*YBX2*	Y-box binding protein 2	CSD	4.22	− 0.38	− 1.06
*ZNF407*	Zinc finger protein 407	zf-C2H2	3.74	0.10	0.95
*ZNF524*	Zinc finger protein 524	zf-C2H2	4.84	0.13	0.73
*PAX7*	Paired box 7	PAX	2.72	− 0.28	0.46
*ZNF326*	Zinc finger protein 326	Others	5.56	0.35	− 0.45
*LBX1*	Ladybird homeobox 1	Homeobox	3.53	0.38	− 0.21
*MAFA*	MAF bZIP transcription factor A	TF_bZIP	3.23	− 0.19	− 0.09
*ZNF146*	Zinc finger protein 146	zf-C2H2	3.46	− 0.20	0.03

^a^The 19 TF belong to 10 different families

^b^The expression average (in log2 CPM) for each TF is indicated by avgexpr variable

### In silico prediction of transcription factor target genes in the post-RIF stage

After the RIF analysis, an in silico prediction was carried out based on the co-expression between each DE TF (n = 19) and its possible DE target genes (n = 274) for each FA condition. Consistently, target genes that were DE between the two conditions were identified (Table 3). PCIT indicated that only six TF genes were significantly correlated with 29 potential targets (*CARHSP1*, *LBX1*, *MAFA*, *PAX7*, *SIX5*, and *TADA2A*). These TF genes belonged to five of the 10 families identified (MYB, CSD, Homeobox, PAX and TF_bZIP).

**Table 3:** In silico prediction of TF target genes for high and low FA profiles, including potential regulators and their putative differentially expressed targets in BC1_DU pigs

Regulatory genes (n = 6)	Number of putative targets	Candidate target genes (n = 29)	
		Condition 1 (less SFA and MUFA, and more PUFA)	Condition 2 (more SFA and MUFA, and less PUFA)
*TADA2A*	15	*TBC1D16*, *TBKBP1*, *WNT5B*, *DHRS3*, *GADD45G*, *KCNT1*, *MAMSTR*, *PLPP7*, *SIX5*	*TFRC*, *APCDD1*, *CARHSP1*, *GOT1*, *PLIN1*, *SOD3*
*CARHSP1*	8	*DNAJB9*, *GOT1*, *MAFA*, *PLIN1*, *TFRC*, *ADIPOQ*	*FBXO6*, *TADA2A*
*SIX5*	5	*TADA2A*, *FBXO6*, *GADD45G*	*FGFR4*, *GALNT15*
*PAX7*	9	*PLPP7*, *TENT5C*, *GALNT15*, *GOT1*, *MAFA*	*WNT5B*, *ADIPOQ*, *FBXO6*, *NEU3*
*MAFA*	8	*PAX7*, *TFRC*, *CARHSP1*, *DHRS3*, *FBXO6*, *FGFR4*, *GADD45G*	*PLIN1*
*LBX1*	3	*FBXO6*, *GALNT15*	*TBKBP1*

Furthermore, as shown in Table 3, the TF genes were positively or negatively correlated with either a specific FA or several FA as shown in Additional file 2: Table S4. Additional information on the distribution of the DE genes and specific TF genes in both conditions and the relationships between log(baseMean) and expression difference are in Additional file 11: Table S9.

Overall, several of the identified candidate target genes (listed in Table 3) are well-known for their role in lipid metabolism (*ADIPOQ*, *DHRS3*, *FGFR4*, *NEU3*, *PLIN1*, *TFRC*, and *WNT5B*), carbohydrate metabolism (*ADIPOQ*, *FBXO6*, *GALNT15*, *GOT1*, *NEU3*, *NUDT14*, and *PPP1R3D*), glucose metabolism (*MAFA*), and ion binding (*ADIPOQ*, *FGFR4*, *GOT1*, *NUDT14*, *SOD3,* and *TADA2A*), among other categories. Thus, this target prediction approach allowed us to uncover target genes of newly identified TF genes in pig muscle, but also target genes that are associated with FA metabolism, such as *TADA2A* and *CARHSP1*. As shown in Table 3, both genes are targets for each other, but also share FA-related target genes (e.g., *GOT1*, *PLIN1*, and *TFRC*).

## Discussion

Our results confirm a complex and bipartite relationship between intramuscular FA composition and gene expression. Gene expression levels can be associated with either specific or several FA traits (either positively or negatively). Our findings of the integrative analysis using FA composition and gene expression datasets complement and extend previous work reported by Valdés-Hernández et al. [9] who performed a univariate association analysis that ignored gene-by-gene interactions and the putative transcriptomic regulators. Here, we analyzed relevant traits such as FA involved in FA ratios that represent enzyme activites. In addition, an updated version of the pig genome annotation was used (release 104 vs. 97). Furthermore, to highlight the most relevant genes and their regulators, a 3-step analytical pipeline was executed, comprising (1) multivariate analysis; (2) partial correlation calculations with information theory (PCIT), and (3) study of RIF associated with FA metabolism. Taken together, the results may facilitate the implementation of breeding strategies based on the use of functional information and improve our understanding of gene regulation in muscle.

### Identification of subsets of canonical variables that maximize the correlation between gene expression and FA profiles, including functional information

Our study provides useful information on representative FA for the SFA, MUFA and PUFA classes, including candidate genes that may be associated with such FA traits. Through exploratory approaches [22, 24–26], we illustrated the relationships between muscle gene expression and intramuscular FA composition. In total, we identified a subset of 378 correlated variables (13 FA and 365 genes).

Interestingly, FA were grouped into two large groups based on the heatmap approach [26]. Essential FA that are obtained only from the diet and their derivative molecules (e.g., C18:2*n*-6, C18:3*n*-3, C20:2*n*-6, C20:3*n*-3, C20:3*n*-6, and C20:4*n*-6) were clustered separately from those that can be derived from biosynthesis (e.g., C14:0, C16:0, C18:0, C18:1*n*-9, and C20:1*n*-9). We also found an interesting inverse relationship between [C20:4*n*-6, C18:3*n*-3] and C18:1*n*-9 FA. This was previously observed in humans, rats, and chickens, which suggests that the inverse association between relative abundances of C18:1*n*-9 (oleic acid) and C20:4*n*-6 (arachidonic acid) is related to C18:3*n*-3 (alpha-linolenic acid) [33] and could reflect the buffering capacity of n-3 FA over inflammatory signals. Omega-6 and omega-3 PUFA have antagonistic inflammatory functions, with C20:4*n*-6 being a pro-inflammatory and immunoactive FA [34] and an important constituent of membrane phospholipids involved in signal transduction [35]. In pigs, carcass fatness shows a positive correlation with IMF. In addition, greater fatness has been associated with a lower relative proportion of PUFA and a higher SFA and MUFA content [36]. Although the n-6/n-3 FA ratio is affected by feeding, for a particular diet, the C18:2*n*-6/C18:3*n*-3 ratio is higher in lean meat compared to meat with a higher fat level [36]. Hence, in the current work, muscle FA composition may be partially explained by differences in IMF (see Additional file 8: Table S6). The oleic acid, C18:1*n*-9, is one of the most predominant MUFA in the triacylglycerols, cholesteryl esters, wax esters, and membrane phospholipids [37], and may also improve meat organoleptic properties and overall acceptability parameters of meat [38].

Analysis of the interaction network [25] revealed the five most interconnected FA (C20:4*n*-6, C18:2*n*-6, C20:3*n*-6, C18.1*n*-9, and C18.0), which had the largest number of associated genes. We found that the *n*-6 FA (C20:4*n*-6, C18:2*n*-6, and C20:3*n*-6) shared more than 51% of its correlated genes (see Additional file 2 Table S4). Remarkably, we also found overlaps of 100% and 80.54% when comparing the C18.1*n*-9 list versus the C20:4*n*-6 and C18:2*n*-6, and against the C20:3*n*-6 list, respectively. It turns out that some of the genes that were correlated with C18.1*n*-9 also displayed other associations, including those with minority FA. This means that C18.1*n*-9 is a key FA in muscle and captures complex associations, with shared or specific genes linked to FA metabolism. Furthermore, five of the 13 analyzed FA (C16:1*n*-7, C20:1*n*-9, C18:0, C18:2*n*-6, and C20:4*n*-6) were correlated with a list of specific genes. For instance, emerging evidence in mice suggests that the lipokine, C16:1*n*-7, is an adipose tissue-derived lipid hormone that strongly stimulates muscle insulin action [39], thereby regulating systemic metabolic homeostasis.

Within the 365 rCCA-derived genes that were grouped into four clusters by the heatmap approach [26], we observed several candidate genes for FA metabolism, which also overlapped with results from previous research in pig populations. Considering the BC1_DU animals, 24 of the 365 FA-correlated genes in LD muscle were identified using a different association analysis strategy [9] (*CPD*, *CYCS*, *LBX1*, *LEP*, *LGALS12*, *LRFN1*, *MAMSTR*, *MDH1*, *NMNAT2*, *NMRK1*, *NUP35*, *OPRL1*, *PDCD1LG2*, *PIK3C2G*, *PLCD3*, *PLIN1*, *PPP1R1B*, *PTPRU*, *SFRP5*, *TENT5C*, *TFRC*, *TIMM8A*, *UNC93A*, and *WNT6*), with overlaps that were mostly observed in only six FA phenotypes (C18:0, C16:1*n*-7, C18:1*n*-9, C20:1*n*-9, C18:3*n*-3, and C20:2*n*-6). Therefore, the genes detected by both strategies pointed to several candidate genes related to FA metabolism, which provided further validation of our findings. Overall, we revealed 10 GO terms and two KEGG pathways that were consistent between the two gene functional studies, which were all mainly related to metabolism and energy homeostasis. Among these, the citrate cycle (TCA cycle or Krebs cycle) is an important aerobic pathway for the final steps of the oxidation of carbohydrates, FA, and amino acids [40], providing precursors for many biosynthetic pathways. For example, common functional genes such as *LEP*, *MDH1*, *CYCS*, and *NMNAT2* were enriched in such GO terms, indicating a potential regulatory role of these genes in FA and energy metabolism.

Although the aforementioned results indicated some degree of overlap in the detected candidate genes and overrepresented GO terms, the univariate association analysis and the integrative analysis used here should be considered as complementary strategies, as they differ in their analytical methods. However, the current study was based on a combination of multivariate methods (i.e. rCCA) [41], which integrates two datasets measured on the same samples (gene expression and FA composition, here without correction for systemic factors). rCCA achieves dimension reduction in each dataset while maximising the similar information between them, thus selecting variables that maximize the correlation. This allows variable selection and priorization at the gene and FA level, with a higher potential to explain the largest proportion of the relationship between the two subsets of data. We conducted a combination of complementary analyses (network, and RIF, in silico prediction of putative target genes, and GO term enrichment) to further prioritize the informative variables and provide insight into biological processes and pathways, in particular among those, the one that is associated with intramuscular FA metabolim.

More specifically, functional analysis with the 365 rCCA-derived genes indicated overrepresentation of the insulin signaling pathway. It is worth noting that the *LEP*, *MDH1*, and *CYCS* genes were also enriched in the insulin signaling pathway, which can affect intramuscular lipid metabolism [42]. Regulation of lipolysis in adipocytes highlights the potential role of certain candidate genes in lipolysis of skeletal muscle (e.g., *PLIN1*) [43], as well as FA derived from intramuscular lipolysis (e.g., C16:1*n*-7 and C18:1*n*-9). In fact, a previous study of porcine adipocytes showed that *PLIN1* (*perilipin 1*) is a novel candidate gene for IMF deposition and adipocyte differentiation [44]. In addition, taking the exemplified FA into account, activation of adipocyte lipolysis by C16:1*n*-7 acid treatment has been demonstrated, while C18:1*n*-9 acid was chosen as a control FA in investigations in mice [45].

Our findings suggest that the six genes that were correlated with FA composition (*ADIPOQ*, *CYP4B1*, *LEP*, *PLIN1*, *SDR16C5*, and *SFRP5*) may also be responsible for IMF deposition [46]. Interestingly, the top 10 highly connected genes with FA (*CYP4B1*, *LEP*, *NUDT14*, *OPRL1*, *PLIN1*, *PPP1R1B*, *PTPRU*, *SFRP5*, *TFRC*, and *UNC93A*) generally showed positive correlations with MUFA and SFA, but negative correlations with PUFA. Therefore, they might be considered as candidate markers of adipocyte physiology. Among these, the *PLIN1*, *LEP,* and *CYP4B1* genes are known to play a pivotal role in the metabolism of FA (FA storage and degradation), while the *UNC93A* gene is related to innate and adaptive immunity [47].

Notably, the gene with the largest number of connections was *PLIN1*, which was associated with 10 of the 13 evaluated FA. With the exception of C18:3*n*-3, four other genes (*LEP*, *PPP1R1B*, *SFRP5*, and *UNC93A*) were connected with the same nine FA as *PLIN1*, presenting a similar pattern of positive correlations with SFA and MUFA, and negative correlations with PUFA. For instance, adipokine genes such as the *LEP* (*leptin*) and *SFRP5* (*secreted frizzled related protein 5*) genes were identified as IMF-correlated genes in the muscle of Duroc × Luchuan pigs [46], and in addition, *SFRP5* has been reported to be significantly DE in the muscle of Duroc pigs with an extreme lipid profile [48]. The *SFRP5* gene encodes an adipokine with anti-inflammatory and insulin-sensitizing properties and appears to have an effect on cytokine release and insulin action in primary adipocytes and skeletal muscle cells [49]. Furthermore, SFRP5 plays an important role in recognizing FA as well as in lipogenesis and, depending on the type of ligand or co-receptor, it can stimulate or inhibit adipogenesis through the WNT pathways [50]. In mice, a long non-coding RNA of the *protein phosphatase 1 regulatory inhibitor subunit 1B* (*PPP1R1B*) gene is involved in skeletal muscle development [51]; such data argue for an important role of the *PPP1R1B*-lncRNA in promoting myogenic differentiation by competing for polycomb repressive complex 2 (PRC2) binding with chromatin of myogenic master regulators. While the relationship between UNC93A (unc-93 homolog A) and the lipid metabolism in muscle has yet to be explored, a study in mice determined that the expression of this putative solute carrier responded to nutrient availability [52]. In addition, *UNC93A* was also mentioned as a candidate gene in a quantitative trait loci (QTL) study for meat quality and disease resistance in the Chinese Jiangquhai pig breed [53].

Apart from the five genes mentioned above, among the 365 rCCA-derived genes several other candidate genes that are involved in lipid metabolism are worth mentioning, such as *ADIPOQ*, *ELOVL6*, *LPIN1*, *G0S2*, *PNPLA8*, and *SOD3.* As another adipokine and candidate for meat quality, the *adiponectin* (*ADIPOQ*) gene was positively correlated with abundance of. C16:1*n*-7. The protein encoded by *ADIPOQ* enhances FA oxidation both in the skeletal muscle and the liver. It stimulates muscle glucose uptake and inhibits glucose production by the liver, thus, decreasing blood glucose levels [54]. Moreover, it has been shown that upregulation of the *ADIPOQ* gene in muscle is associated with inhibition of fat deposition in castrated male Iberian pigs (Torbiscal variety) [55].

For regulation of lipogenesis, the *ELOVL fatty acid elongase 6* (*ELOVL6*) gene is of particular interest, as it is directly involved in elongation of FA in mammals [56]. In our group, Corominas et al. [57] previously reported a plausible effect of the expression levels of *ELOVL6* on the abundance of C16:0 and C16:1*n*-7 FA in backfat and muscle of Landrace backcross pigs. However, in our study, the BC1_DU pigs showed only a negative correlation with abundance of the essential FA C18:2*n*-6. As *ELOVL6* does not elongate C18:2*n*-6, this negative correlation may be due to the increase in the relative abundance of other SFA and MUFA caused by *ELOVL6*. Regarding regulation of adipogenesis, our data revealed that the *lipin 1* (*LPIN1*) gene was positively correlated with abundance of C20:4*n*-6. This gene has been shown to participate in metabolism of the arachidonic acid in *Caenorhabditis elegans* [58]. Furthermore, in a previous study from our group, *LPIN1* has also been investigated as a potential candidate gene for intramuscular FA composition in a Landrace backcross population [4].

Apart from *PLIN1*, other genes involved in lipolysis and/or adipogenesis were *FBP1*, *G0S2,* and *SOD3*, which were positively correlated with abundance of C18:1*n*-9 and negatively correlated with abundances of C18:2*n*-6, C20:3*n*-6, and C20:4*n*-6. For instance, the expression level of the porcine *G0/G1 switch 2* (*G0S2*) gene increased significantly during adipogenesis (both in in vitro and in vivo studies) [59]. In the same study, G0S2 was suggested to be a negative regulator of adipose triglyceride lipase (ATGL)-mediated lipolysis and cell proliferation in adipose tissue, and to be closely related to lipid accumulation [59]. As an antioxidant enzyme, SOD3 (superoxide dismutase 3) is secreted by adipocytes and has the potential to prevent oxidative stress. In mice, Cui et al. [60] suggested that SOD3 had a specific function in blocking adipogenesis and that overexpression of *SOD3* suppressed expression of pro-inflammatory genes in adipose tissue, and increased expression of anti-inflammatory genes.

The positive correlations of levels of expression of the *FBP1*, *G0S2,* and *SOD3* genes with abundance of oleic acid (C18:1*n*-9), and their negative correlations with the three other PUFA may be due to their involvement in lipid metabolism of the host, rather than to metabolism of the essential FA and their derivatives. In fact, we highlighted candidate genes, such as *ADIPOQ*, *ELOVL6*, and *PLIN1* that were also identified as overexpressed genes in individuals that were divergent for IMF values and had a higher IMF content according to transcriptome analyses of LD muscle in Iberian pigs [61]. Furthermore, Benítez et al. [62] found that breed (Iberian/Duroc) had a modulatory effect on the expression of the *ELOVL6* and *LEP* genes (adjusted *P*-value < 0.10) in the adipose tissue from growing pigs; lipogenic (*ELOVL6* and *LEP*) and lipolytic (*G0S2* and *PLIN1*) genes had a higher expression in biopsies obtained from the Iberian pigs. This seems to be the case when lipid deposition in the muscle of pigs is the result of a balance between lipogenesis and lipolysis processes [63]. Nevertheless, fat accumulation in animals results from an imbalance between synthesis and degradation. When synthesis of FA is greater than their consumption, FA are deposited in cells instead of mobilized to provide energy [64]. In addition, in an association study for backfat FA composition in free-range Iberian pigs, two single nucleotide polymorphisms (SNPs) *ADIPOQ*:g.124646194T>G and *ELOVL6*:g.112186423A>G were identified to have a significant association [65].

We also found that the levels of expression of genes such as *patatin-like phospholipase domain containing 8* (*PNPLA8*), *glutamate oxaloacetate transaminase 1* (*GOT1*), and *3-hydroxy-3-methylglutaryl-CoA synthase 1* (*HMGCS1*) were negatively correlated with abundance of C18:1*n*-9, while they were positively correlated with abundance of C18:2*n*-6 and C20:4*n*-6, together with the level of expression of the *3-hydroxy-3-methylglutaryl-CoA reductase* (*HMGCR*) gene. The PNPLA8 protein (also known as iPLA2γ) plays an important role in lipolysis and FA oxidation. It belongs to a family of phospholipases that catalyze the cleavage of FA from membrane phospholipids [66]. Interestingly, PNPLA8 may preferentially act on arachidonic containing membrane phospholipids (C20:4*n*-6, a FA that can undergo beta-oxidation) to generate free arachidonic acid, along with lysophosphatidic acid [67]. Therefore, PNPLA8 plays an important role in mobilization of arachidonic acid in response to cellular stimuli [68] and in release of lipid second messengers. As another candidate for meat quality and carbohydrate metabolism, the *GOT1* gene controls cellular metabolism by coordinating utilization of carbohydrates and amino acids to meet nutrient requirements [69], but it is also crucial in providing oxaloacetate at low glucose levels, likely to maintain the redox homeostasis. In contrast, the *HMGCR* gene encodes a cholesterol-synthesis limiting enzyme, an enzyme of the mevalonate pathway, which participates in fat deposition and is associated with meat composition traits [70, 71]. In addition, synonymous polymorphisms in this gene, such as *HMGCR*:c.807A>C, have been shown to be associated with muscle lipid deposition and cholesterol-related traits in Duroc pigs with an extreme lipid profile [70].

It is also worth mentioning that the two transporter genes *exocyst complex component 7* (*EXOC7*) and *solute carrier family 44 member 2* (*SLC44A2*) may have a role in lipid metabolism. The level of expression of the *EXOC7* gene was found to be positively correlated with abundance of C18:0. As EXOC7 is a component of the exocyst complex, which regulates free FA uptake by adipocytes [72], it is involved in diverse biological functions, including promoting translocation of the glucose transporter GLUT4 in the cell. Although there are other members of the solute carrier family, the *SLC44A2* gene presented the same correlation pattern (positive with MUFA but negative with PUFA) as the *FBP1*, *G0S2* and *SOD3* genes. In mice, SLC44A2 mediates choline transport into mitochondria, and regulates synthesis of ATP, platelet activation and thrombosis [73]. In addition, supporting information for *SLC44A2* has suggested its important role for normal homeostasis [74].

### Identification of potential regulators of gene expression and their putative gene targets in muscle

We used RIF analysis to highlight putative regulators and to assess their potential role in controlling their predicted target genes. We identified six TF-regulator genes, including two novel (*TADA2A* and *CARHSP1*) but also four well-documented regulators (*MAFA*, *SIX5*, *LBX1,* and *PAX7*). Remarkably, among these six TF genes, *TADA2A* and *CARSHP1* based on RIF1 and *KLF10* and *TADA2A* based on RIF2 were scored as the first and second most relevant TF genes. In addition, the *LBX1*, *KLF10*, *PAX7*, and *SIX5* genes encode TF that may be putative regulators of lean muscle growth. Moreover, several target genes of TADA2A and CARSHP1 were detected as being functionally related to lipid metabolism (e.g., *PLIN1* and *TFRC*) and/or meat quality (e.g., *GOT1*). However, no target genes were detected for KLF10.

Considering the rCCA approach, our findings in muscle suggest that the *transcriptional adaptor 2A* (*TADA2A*) gene was linked to the four most interconnected FA (positively associated with C20:4*n*-6, C18:2*n*-6, and C20:3*n*-6, respectively, and negatively associated with C18:1*n*-9). The TADA2A protein is part of the ATAC (Ada-Two-A-Containing) complex that interacts with the TATA-binding protein for transcriptional activation [75]. In addition, *TADA2A* has been suggested to be involved in de novo hepatic lipogenesis in chickens fed different diets [76]. The level of expression of the *calcium regulated heat stable protein 1* (*CARHSP1*) gene was positively linked to the sixth most interconnected FA (C16:1*n*-7). In mice, *CARHSP1* is regulated by the nutrient status in the liver and was suggested to inhibit hepatic gluconeogenic gene expression via repression of the transcriptional activity of the *PPARα* transcription factor [77]. Consequently, these two regulators were targets for each other in animals with condition 2 (i.e., more SFA and MUFA but fewer PUFA, and higher IMF content), as well as having shared target genes, such as *GOT1*, *PLIN1,* and *TFRC*. Taken together, these findings point to some of the potential transcriptional circuits through which key regulatory genes exert their impact on their targets and FA. Conversely, FA may also act as signaling candidates to regulate transcription of target genes that encode proteins that are involved in muscle lipid metabolism [78]. In turn, gene expression may also be modulated by FA abundance, as suggested by independent studies that correlate gene expression with FA composition or with IMF content in pig muscle [1, 4, 9, 46, 79, 80]. Other factors can also affect gene expression, such as environmental factors, genetic background, breeding systems, management, and host-factors. It has been reported that the FA composition in pig diets affects subcutaneous IMF FA profile [64, 79]. Recently, Ludwiczak et al. [81] also pointed out that FA profiles (SFA, MUFA, and PUFA) in the loin (e.g., in *longissimus thoracis et lumborum* muscle) or IMF content of European pigs (e.g., Nero Siciliano, Cinta Sense, and Iberian × Duroc) can be affected by diet or by the interaction between diet and housing system. However, in the present work a uniform diet was provided to all animals.

## Conclusions

The findings of this study contribute to a better understanding of the complex relationship between FA composition and gene expression in muscle, but may also reveal patterns of gene expression involving Iberian and Duroc pigs. Based on the results of the rCCA, functional analysis, RIF analysis, prediction of target genes, and supporting literature, all the genes discussed above are promising candidates for muscle lipid deposition and FA composition in Iberian and Duroc pigs. Our rCCA-derived findings highlighted genes encoding enzymes associated with fat deposition, but also bioprocesses and metabolic pathways involved in the determination of FA traits. Using a complementary RIF analysis, we proposed two novel regulators (TADA2A and CARHSP1) for intramuscular FA metabolism. Functional analyses at the GO and pathway levels reinforced the significance of biological processes associated with energy, lipid, and carbohydrate metabolism, as well as of the KEGG pathway of regulation of lipolysis in adipocytes in muscle tissue. Furthermore, our results highlighted the *ADIPOQ, ELOVL6, G0S2, HMGCR, LEP, LPIN1, PLIN1, PNPLA8,* and *SFRP5* genes for their lipogenic and/or lipolytic potential to contribute to intramuscular FA composition. Our study also identified the endogenous antioxidant enzyme SOD3 as having a promising role in regulation of adipogenesis in pig muscle.

### Supplementary Information

**Additional file 1: Table S1.** Title: Manual list with functional annotation and plausible function in different aspects of the FA, lipid, and carbohydrate metabolism. Description: This list was compiled based on ClueGO results for selected genes from rCCA. **Table S2.** Title: Trained list containing genes associated with lipid and/or carbohydrate metabolism in lipogenic tissues (adipose, liver and muscle-skeletal) and obtained from the GUILDify tool. Description: This procedure uses the BIANA knowledge database to create a species-specific interaction network for each gene detected. Here, the netcombo prioritization algorithm based on network topology, and the highest guild score for the top 100 gene products (with only seed) were considered to constitute the trained list. **Table S3.** Title: List of all transcription factors (TF) and TF cofactors for the *Sus scrofa* specie obtained from the AnimalTFDB v3.0 database. Description: This resource is also available at: http://bioinfo.life.hust.edu.cn/AnimalTFDB/#!/.

**Additional file 2: Table S4.** Title: The correlation matrix for all bipartite relationships (13 FA and 365 genes) obtained via the rCCA approach with a correlation above 0.29 using the network function in the mixOmics package. Description: NA values in this table mean that the correlation between both variables did not exceed the pre-established cut-off point.

**Additional file 3: Figure S1.** Title: Correlation circle plot from the PCA applied to the FA phenotypes and gene expression in muscle of BC1_DU pigs displayed the first versus third rCCA dimensions. Description: This output was obtained using the plotVar function of the mixOmics package.

**Additional file 4: Figure S2.** Title: Heatmap displaying the correlation structure of the rCCA in the *longissimus dorsi* muscle from BC1_DU pigs. Description: This output was obtained using the functions and dependencies of the ComplexHeatmap package. All bipartite relationships between FA and gene expression variables (cutoff of 0.29) are shown, including hierarchical clusters for both variables. Heatmap with the rCCA variables (365 genes and 13 FA selected in total) was computed. To complement the network plot, heatmap was used. The color key indicates positive (green) and negative (red) correlation.

**Additional file 5: Figure S3.** Title: Density distribution of correlation between FA and gene expression profiles, including five quantile levels and mean value in the *longissimus dorsi* muscle from BC1_DU pigs. Description: Distribution of correlations (FA vs. gene expression) as density heatmap using the *densityHeatmap* function. Here, the density was calculated by column from input data passed as a list item.

**Additional file 6: Figure S4.** Title: Functional analysis with correlated genes from rCCA that were significantly enriched in GO terms according to delimitation of biological processes. Description: This output is a representation of the original table of results generated with the ClueGO plugin in the Cytoscape software.

**Additional file 7: Table S5.** Title: ClueGO Gene Ontology (GO) results of selected genes in the rCCA approach. Description: Functional analysis included significant GO terms with and without “GO Term Fusion” (biological process, pathways and molecular functions, BH-corrected *P*-value < 0.05) and was performed with the background of all genes expressed in muscle.

**Additional file 8: Table S6.** Title: Mean comparison between the two FA conditions with the extreme values obtained through classification using PCA (see details in Fig. 4). Description: This analysis included the FA profile (n = 60 animals) with the 13 phenotypes selected in the rCCA approach, and in addition the IMF content. Significant differences (*P*-value < 0.05) between FA conditions were determined using a t-test, and the standard error of the mean (SEM) was calculated.

**Additional file 9: Table S7.** Title: Results of the Pearson correlation between each pair of FA (n = 15) using 60 samples delimited in two conditions. Description: This output was obtained using the *cor.test* function using R base.

**Additional file 10: Table S8.** Title: Results of the analysis of differentially expressed (DE) genes between the two FA conditions (lfc = 1.5 and padj = 0.05). Description: This output was obtained using the functions of the CeTF package.

**Additional file 11: Table S9.** Title: Results of the distribution of DE genes and key TF in both conditions, as well as the relationship between log(baseMean) and expression difference. Description: This output was obtained using the functions of the CeTF package.

## Data Availability

All relevant data produced or evaluated in this research are disclosed in the paper as well as its supplementary information files. The RNA sequencing data used and analyzed in the current study are available from sequence read archive (SRA), NCBI BioProject under the accession number PRJNA882638 (https://www.ncbi.nlm.nih.gov/sra).

## References

[1] Zhang J, Cui L, Ma J, Chen C, Yang B, Huang L (2017). Transcriptome analyses reveal genes and pathways associated with fatty acid composition traits in pigs. Anim Genet.

[2] Wood JD, Richardson RI, Nute GR, Fisher AV, Campo MM, Kasapidou E (2004). Effects of fatty acids on meat quality: a review. Meat Sci.

[3] Storrustløkken L, Ekeberg D, Egelandsdal B, Håseth TT, Alvseike O (2014). Effect of intramuscular fat, breed, and age at slaughter on fatty acid composition in green hams. J Food Sci.

[4] Puig-Oliveras A, Revilla M, Castelló A, Fernández AI, Folch JM, Ballester M (2016). Expression-based GWAS identifies variants, gene interactions and key regulators affecting intramuscular fatty acid content and composition in porcine meat. Sci Rep.

[5] Ramayo-Caldas Y, Mach N, Esteve-Codina A, Corominas J, Castelló A, Ballester M (2012). Liver transcriptome profile in pigs with extreme phenotypes of intramuscular fatty acid composition. BMC Genomics.

[6] Corominas J, Ramayo-Caldas Y, Puig-Oliveras A, Estellé J, Castelló A, Alves E (2013). Analysis of porcine adipose tissue transcriptome reveals differences in de novo fatty acid synthesis in pigs with divergent muscle fatty acid composition. BMC Genomics.

[7] Puig-Oliveras A, Ramayo-Caldas Y, Corominas J, Estellé J, Pérez-Montarelo D, Hudson NJ (2014). Differences in muscle transcriptome among pigs phenotypically extreme for fatty acid composition. PLoS One..

[8] Ropka-Molik K, Zukowski K, Eckert R, Gurgul A, Piõrkowska K, Oczkowicz M (2014). Comprehensive analysis of the whole transcriptomes from two different pig breeds using RNA-Seq method. Anim Genet.

[9] Valdés-Hernández J, Ramayo-Caldas Y, Passols M, Sebastià C, Criado-Mesas L, Crespo-Piazuelo D (2023). Global analysis of the association between pig muscle fatty acid composition and gene expression using RNA-Seq. Sci Rep.

[10] Rohart F, Gautier B, Singh A, Lê Cao KA (2017). mixOmics: an R package for ‘omics feature selection and multiple data integration. PLoS Comput Biol.

[11] Cesar ASM, Regitano LCA, Koltes JE, Fritz-Waters ER, Lanna DPD, Gasparin G (2015). Putative regulatory factors associated with intramuscular fat content. PLoS One..

[12] Reverter A, Hudson NJ, Nagaraj SH, Pérez-Enciso M, Dalrymple BP (2010). Regulatory impact factors: unraveling the transcriptional regulation of complex traits from expression data. Bioinformatics.

[13] Martínez-Montes ÁM, Fernández A, Muñoz M, Noguera JL, Folch JM, Fernández AI (2018). Using genome wide association studies to identify common QTL regions in three different genetic backgrounds based on Iberian pig breed. PLoS One..

[14] Mach N, Devant M, Díaz I, Font-Furnols M, Oliver MA, García JA (2006). Increasing the amount of n-3 fatty acid in meat from young Holstein bulls through nutrition. J Anim Sci.

[15] Crespo-Piazuelo D, Criado-Mesas L, Revilla M, Castelló A, Noguera JL, Fernández AI (2020). Identification of strong candidate genes for backfat and intramuscular fatty acid composition in three crosses based on the Iberian pig. Sci Rep.

[16] Babraham Bioinformatics-FastQC a quality control tool for high throughput sequence data. 2019. https://www.bioinformatics.babraham.ac.uk/projects/fastqc. Accessed 01 Aug 2019.

[17] Ewels P, Magnusson M, Lundin S, Käller M (2016). MultiQC: summarize analysis results for multiple tools and samples in a single report. Bioinformatics.

[18] Dobin A, Davis CA, Schlesinger F, Drenkow J, Zaleski C, Jha S (2013). STAR: ultrafast universal RNA-seq aligner. Bioinformatics.

[19] Li B, Dewey CN (2011). RSEM: accurate transcript quantification from RNA-Seq data with or without a reference genome. BMC Bioinformatics.

[20] Robinson MD, McCarthy DJ, Smyth GK (2010). edgeR: a bioconductor package for differential expression analysis of digital gene expression data. Bioinformatics.

[21] Durinck S, Spellman PT, Birney E, Huber W (2009). Mapping identifiers for the integration of genomic datasets with the R/Bioconductor package biomaRt. Nat Protoc.

[22] Lê Cao KA, González I, Déjean S (2009). integrOmics: an R package to unravel relationships between two omics datasets. Bioinformatics.

[23] Ramayo-Caldas Y, Mármol-Sánchez E, Ballester M, Sánchez JP, González-Prendes R, Amills M (2019). Integrating genome-wide co-association and gene expression to identify putative regulators and predictors of feed efficiency in pigs. Genet Sel Evol.

[24] Csardi G, Nepusz T (2006). The igraph software package for complex network research. InterJournal.

[25] Shannon P, Markiel A, Ozier O, Baliga NS, Wang JT, Ramage D (2003). Cytoscape: a software environment for integrated models of biomolecular interaction networks. Genome Res.

[26] Gu Z, Eils R, Schlesner M (2016). Complex heatmaps reveal patterns and correlations in multidimensional genomic data. Bioinformatics.

[27] Bindea G, Mlecnik B, Hackl H, Charoentong P, Tosolini M, Kirilovsky A (2009). ClueGO: a Cytoscape plug-in to decipher functionally grouped gene ontology and pathway annotation networks. Bioinformatics.

[28] Benjamini Y, Hochberg Y (1995). Controlling the false discovery rate: a practical and powerful approach to multiple testing. R Stat Soc Series B Stat Methodol.

[29] Wickham H (2016). ggplot2: elegant graphics for data analysis.

[30] Oliveira de Biagi CA, Nociti RP, Brotto DB, Funicheli BO, CássiaRuy de P, Bianchi Ximenez JP (2021). CeTF: an R/bioconductor package for transcription factor co-expression networks using regulatory impact factors (RIF) and partial correlation and information (PCIT) analysis. BMC Genomics..

[31] Kassambara A, Mundt F. factoextra: extract and visualize the results of multivariate data analyses. 2020. https://cran.r-project.org/package=factoextra. Accessed 01 Apr 2020.

[32] Guney E, Garcia-Garcia J, Oliva B (2014). GUILDify: a web server for phenotypic characterization of genes through biological data integration and network-based prioritization algorithms. Bioinformatics.

[33] Høstmark AT, Haug A (2014). The inverse association between relative abundances of oleic acid and arachidonic acid is related to alpha-linolenic acid. Lipids Health Dis.

[34] Wall R, Ross RP, Fitzgerald GF, Stanton C (2010). Fatty acids from fish: the anti-inflammatory potential of long-chain omega-3 fatty acids. Nutr Rev.

[35] Spector AA (1999). Essentiality of fatty acids. Lipids.

[36] De Smet S, Raes K, Demeyer D (2004). Meat fatty acid composition as affected by fatness and genetic factors: a review. Anim Res.

[37] O’Neill LM, Miyazaki M, Bond LM, Lewis SA, Ding F, Liu Z, Ridgway ND, McLeod RS (2021). Fatty acid desaturation and elongation in mammals. Biochemistry of lipids, lipoproteins and membranes.

[38] Cameron ND, Enser M, Nute GR, Whittington FM, Penman JC, Fisken AC (2000). Genotype with nutrition interaction on fatty acid composition of intramuscular fat and the relationship with flavour of pig meat. Meat Sci.

[39] Cao H, Gerhold K, Mayers JR, Wiest MM, Watkins SM, Hotamisligil GS (2008). Identification of a lipokine, a lipid hormone linking adipose tissue to systemic metabolism. Cell.

[40] Akram M (2014). Citric acid cycle and role of its intermediates in metabolism. Cell Biochem Biophys.

[41] Lê Cao K-A, Welham Z (2022). Multivariate data integration using R: methods and applications with the mixOmics package.

[42] Consitt LA, Bell JA, Houmard JA (2009). Intramuscular lipid metabolism, insulin action, and obesity. IUBMB Life.

[43] MacPherson REK, Peters SJ (2015). Piecing together the puzzle of perilipin proteins and skeletal muscle lipolysis. Appl Physiol Nutr Metab.

[44] Li B, Weng Q, Dong C, Zhang Z, Li R, Liu J (2018). A key gene, *PLIN1*, can affect porcine intramuscular fat content based on transcriptome analysis. Genes (Basel).

[45] Bolsoni-Lopes A, Festuccia WT, Farias TSM, Chimin P, Torres-Leal FL, Derogis PBM (2013). Palmitoleic acid (n-7) increases white adipocyte lipolysis and lipase content in a PPARα-dependent manner. Am J Physiol Endocrinol Metab.

[46] Liu Y, Long H, Feng S, Ma T, Wang M, Niu L (2021). Trait correlated expression combined with eQTL and ASE analyses identified novel candidate genes affecting intramuscular fat. BMC Genomics.

[47] Kim TH, Kim D, Lee Y, Kwon HJ (2014). Expression of UNC93A induced by CpG-DNA-liposome complex in mice. J Korean Soc Appl Biol Chem.

[48] Cardoso TF, Cánovas A, Canela-Xandri O, González-Prendes R, Amills M, Quintanilla R (2017). RNA-seq based detection of differentially expressed genes in the skeletal muscle of Duroc pigs with distinct lipid profiles. Sci Rep.

[49] Carstensen M, Wiza C, Röhrig K, Fahlbusch P, Roden M, Herder C (2014). Effect of Sfrp5 on cytokine release and insulin action in primary human adipocytes and skeletal muscle cells. PLoS One..

[50] Chu DT, Nguyen TL (2023). Frizzled receptors and SFRP5 in lipid metabolism: current findings and potential applications. Prog Mol Biol Transl Sci.

[51] Kang X, Zhao Y, Van Arsdell G, Nelson SF, Touma M (2020). Ppp1r1b-lncRNA inhibits PRC2 at myogenic regulatory genes to promote cardiac and skeletal muscle development in mouse and human. RNA.

[52] Ceder MM, Lekholm E, Hellsten SV, Perland E, Fredriksson R (2017). The neuronal and peripheral expressed membrane-bound UNC93A respond to nutrient availability in mice. Front Mol Neurosci.

[53] Oyelami FO, Zhao Q, Xu Z, Zhang Z, Sun H, Zhang Z (2020). Haplotype block analysis reveals candidate genes and QTLs for meat quality and disease resistance in Chinese Jiangquhai pig breed. Front Genet.

[54] Karbowska J, Kochan Z (2006). Role of adiponectin in the regulation of carbohydrate and lipid metabolism. J Physiol Pharmacol.

[55] Villaplana-Velasco A, Noguera JL, Pena RN, Ballester M, Muñoz L, González E (2021). Comparative transcriptome profile between Iberian pig varieties provides new insights into their distinct fat deposition and fatty acids content. Animals (Basel).

[56] Jakobsson A, Westerberg R, Jacobsson A (2006). Fatty acid elongases in mammals: their regulation and roles in metabolism. Prog Lipid Res.

[57] Corominas J, Ramayo-Caldas Y, Puig-Oliveras A, Pérez-Montarelo D, Noguera JL, Folch JM (2013). Polymorphism in the ELOVL6 gene is associated with a major QTL effect on fatty acid composition in pigs. PLoS One..

[58] Jung Y, Kwon S, Ham S, Lee D, Park HH, Yamaoka Y (2020). *Caenorhabditis elegans* Lipin 1 moderates the lifespan-shortening effects of dietary glucose by maintaining ω-6 polyunsaturated fatty acids. Aging Cell.

[59] Ahn J, Oh SA, Suh Y, Moeller SJ, Lee K (2013). Porcine *G0/G1 switch gene 2* (*G0S2*) expression is regulated during adipogenesis and short-term in-vivo nutritional interventions. Lipids.

[60] Cui R, Gao M, Qu S, Liu D (2014). Overexpression of superoxide dismutase 3 gene blocks high-fat diet-induced obesity, fatty liver and insulin resistance. Gene Ther.

[61] Muñoz M, García-Casco JM, Caraballo C, Fernández-Barroso MÁ, Sánchez-Esquiliche F, Gómez F (2018). Identification of candidate genes and regulatory factors underlying intramuscular fat content through longissimus dorsi transcriptome analyses in heavy Iberian pigs. Front Genet.

[62] Benítez R, Fernández A, Isabel B, Núñez Y, De Mercado E, Gómez-Izquierdo E (2017). Modulatory effects of breed, feeding status, and diet on adipogenic, lipogenic, and lipolytic gene expression in growing Iberian and Duroc pigs. Int J Mol Sci.

[63] Pena RN, Quintanilla R, Manunza A, Gallardo D, Casellas J, Amills M (2014). Application of the microarray technology to the transcriptional analysis of muscle phenotypes in pigs. Anim Genet.

[64] Yi W, Huang Q, Wang Y, Shan T (2023). Lipo-nutritional quality of pork: the lipid composition, regulation, and molecular mechanisms of fatty acid deposition. Anim Nutr.

[65] Palma-Granados P, García-Casco JM, Caraballo C, Vázquez-Ortego P, Gómez-Carballar F, Sánchez-Esquiliche F (2023). Design of a low-density SNP panel for intramuscular fat content and fatty acid composition of backfat in free-range Iberian pigs. Anim Sci..

[66] Stelzer G, Rosen N, Plaschkes I, Zimmerman S, Twik M, Fishilevich S (2016). The GeneCards suite: from gene data mining to disease genome sequence analyses. Curr Protoc Bioinformatics..

[67] Kim KY, Jang HJ, Yang YR, Park K, Seo JK, Shin IW (2016). SREBP-2/PNPLA8 axis improves non-alcoholic fatty liver disease through activation of autophagy. Sci Rep..

[68] Yan W, Jenkins CM, Han X, Mancuso DJ, Sims HF, Yang K (2005). The highly selective production of 2-arachidonoyl lysophosphatidylcholine catalyzed by purified calcium-independent phospholipase A2γ: identification of a novel enzymatic mediator for the generation of a key branch point intermediate in eicosanoid signaling. J Biol Chem.

[69] Zhou X, Curbo S, Li F, Krishnan S, Karlsson A (2018). Inhibition of glutamate oxaloacetate transaminase 1 in cancer cell lines results in altered metabolism with increased dependency of glucose. BMC Cancer.

[70] Cánovas A, Quintanilla R, Gallardo D, Díaz I, Noguera JL, Ramírez O (2010). Functional and association studies on the pig HMGCR gene, a cholesterol-synthesis limiting enzyme. Animal.

[71] Brown MS, Goldstein JL (1980). Multivalent feedback regulation of HMG CoA reductase, a control mechanism coordinating isoprenoid synthesis and cell growth. J Lipid Res.

[72] Inoue M, Akama T, Jiang Y, Chun TH (2015). The exocyst complex regulates free fatty acid uptake by adipocytes. PLoS One..

[73] Bennett JA, Mastrangelo MA, Ture SK, Smith CO, Loelius SG, Berg RA (2020). The choline transporter Slc44a2 controls platelet activation and thrombosis by regulating mitochondrial function. Nat Commun.

[74] Nair TS, Kommareddi PK, Galano MM, Miller DM, Kakaraparthi BN, Telian SA (2016). *SLC44A2* single nucleotide polymorphisms, isoforms, and expression: association with severity of Meniere’s disease?. Genomics.

[75] Wang YL, Faiola F, Xu M, Pan S, Martinez E (2008). Human ATAC is a GCN5/PCAF-containing acetylase complex with a novel NC2-like histone fold module that interacts with the TATA-binding protein. J Biol Chem.

[76] Desert C, Baéza E, Aite M, Boutin M, Le Cam A, Montfort J (2018). Multi-tissue transcriptomic study reveals the main role of liver in the chicken adaptive response to a switch in dietary energy source through the transcriptional regulation of lipogenesis. BMC Genomics.

[77] Fan Y, Guo Y, Hamblin M, Chang L, Zhang J, Chen YE (2011). Inhibition of gluconeogenic genes by calcium-regulated heat-stable protein 1 via repression of peroxisome proliferator-activated receptor α. J Biol Chem.

[78] Fritzen AM, Lundsgaard AM, Kiens B (2020). Tuning fatty acid oxidation in skeletal muscle with dietary fat and exercise. Nat Rev Endocrinol.

[79] Wang H, Wang J, Yang DD, Liu ZL, Zeng YQ, Chen W (2020). Expression of lipid metabolism genes provides new insights into intramuscular fat deposition in Laiwu pigs. Asian-Australas J Anim Sci.

[80] Wang W, Xue W, Jin B, Zhang X, Ma F, Xu X (2013). Candidate gene expression affects intramuscular fat content and fatty acid composition in pigs. J Appl Genet.

[81] Ludwiczak A, Kasprowicz-Potocka M, Zaworska-Zakrzewska A, Składanowska-Baryza J, Rodriguez-Estevez V, Sanz-Fernandez S (2023). Husbandry practices associated with extensification in European pig production and their effects on pork quality. Meat Sci.

