# Correction: Identification of candidate regulatory genes for intramuscular fatty acid composition in pigs by transcriptome analysis

**DOI:** 10.1186/s12711-024-00885-8

**Published:** 2024-02-26

**Authors:** Jesús Valdés-Hernández, Josep M. Folch, Daniel Crespo-Piazuelo, Magí Passols, Cristina Sebastià, Lourdes Criado-Mesas, Anna Castelló, Armand Sánchez, Yuliaxis Ramayo-Caldas

**Affiliations:** 1 Plant and Animal Genomics, Centre for Research in Agricultural Genomics (CRAG), CSIC-IRTA-UAB-UB, Campus UAB, Bellaterra, Spain; 2 Departament de Ciència Animal i dels Aliments, Facultat de Veterinària, Universitat Autònoma de Barcelona, Bellaterra, Spain; 3 Departament de Genètica i Millora Animal, Institut de Recerca y Tecnologia Agraroalimentàries (IRTA), Caldes de Montbui, Spain

**Correction: Genetics Selection Evolution (2024) 56:12** 10.1186/s12711-024-00882-x

After publication of original article [1], we noticed that two errors were introduced during production:

1. In the **Bioinformatic and statistical analyses** section, the corresponding information on the **X** and **Y** matrices has been removed in three places:
  The part “A regularized canonical correlation analysis (rCCA) was performed using the expression dataset of the 12,381 genes (matrix) and the 15 FA traits (matrix) measured on the 129 individuals. The rCCA multivariate approach is implemented in the mixOmics v6.14.1 package [12], which allows subsets of canonical variables that maximize the correlation between two datasets (“and”, respectively of sizes n × p and n × q) to be identified [22].”
  Should be “A regularized canonical correlation analysis (rCCA) was performed using the expression dataset of the 12,381 genes (matrix **Y**) and the 15 FA traits (matrix **X**) measured on the 129 individuals. The rCCA multivariate approach is implemented in the mixOmics v6.14.1 package [10], which allows to identify the subsets of canonical variables that maximize the correlation between two datasets (“**X** and **Y**”, respectively of sizes n × p and n × q) [22].”
2. In the Funding section, the following term “https://doi.org/” has been automatically added to the funding source in three places. In fact, the correct funding source should be “MCIN/AEI/10.13039/501100011033” and not “MCIN/AEI/https://doi.org/10.13039/501100011033”.
