# Supplementary figures and images for: Identification of candidate regulatory genes for intramuscular fatty acid composition in pigs by transcriptome analysis
Source: Genet Sel Evol. 2024 Feb 12;56:12. doi: 10.1186/s12711-024-00882-x (PMC10860264; doi:10.1186/s12711-024-00882-x)

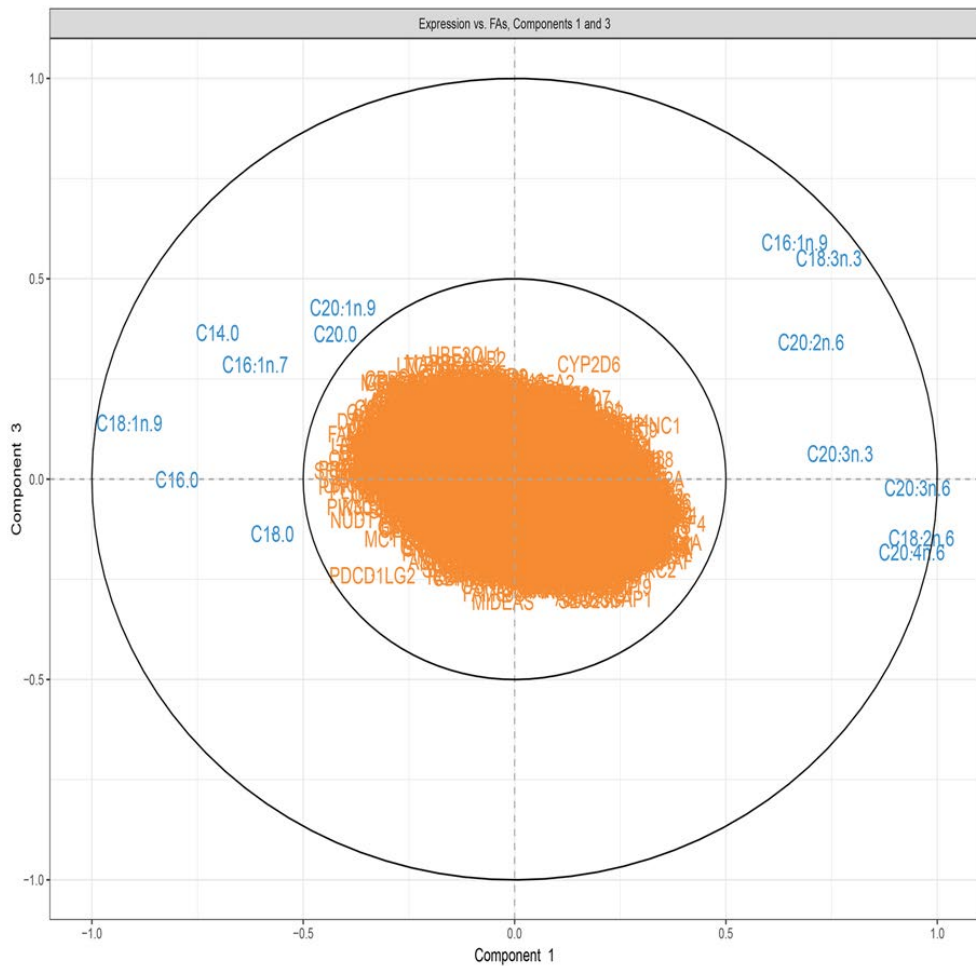

Supplement: Supplementary file 3 — Additional file 3: Figure S1. Title: Correlation circle plot from the PCA applied to the FA phenotypes and gene expression in muscle of BC1_DU pigs displayed the first versus third rCCA dimensions. Description: This output was obtained using the plotVar function of the mixOmics package. [file 12711_2024_882_MOESM3_ESM.pdf]

1

2

3

4

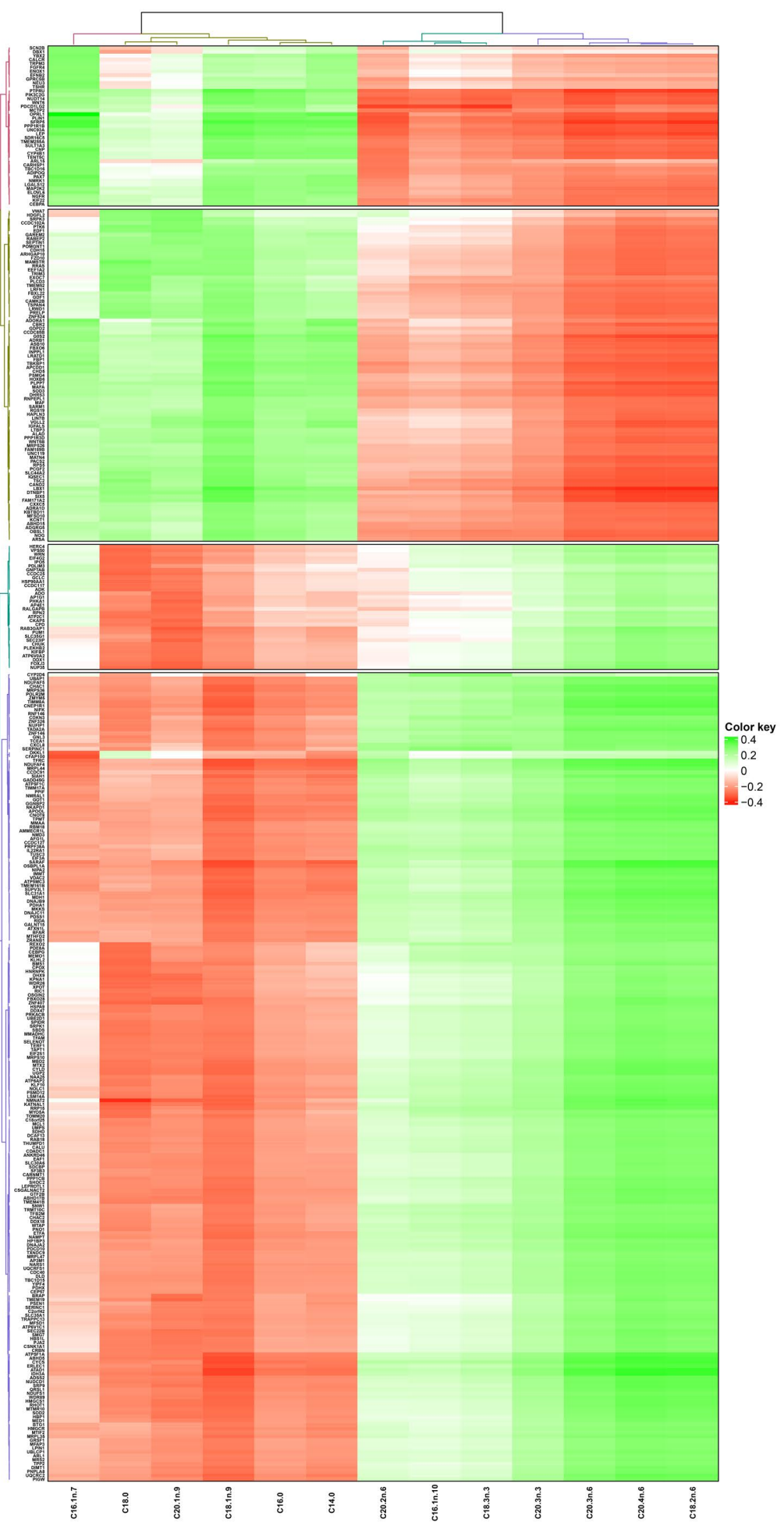

Supplement: Supplementary file 4 — Additional file 4: Figure S2. Title: Heatmap displaying the correlation structure of the rCCA in the longissimus dorsi muscle from BC1_DU pigs. Description: This output was obtained using the functions and dependencies of the ComplexHeatmap package. All bipartite relationships between FA and gene expression variables (cutoff of 0.29) are shown, including hierarchical clusters for both variables. Heatmap with the rCCA variables (365 genes and 13 FA selected in total) was computed. To complement the network plot, heatmap was used. The color key indicates positive (green) and negative (red) correlation. [file 12711_2024_882_MOESM4_ESM.pdf]

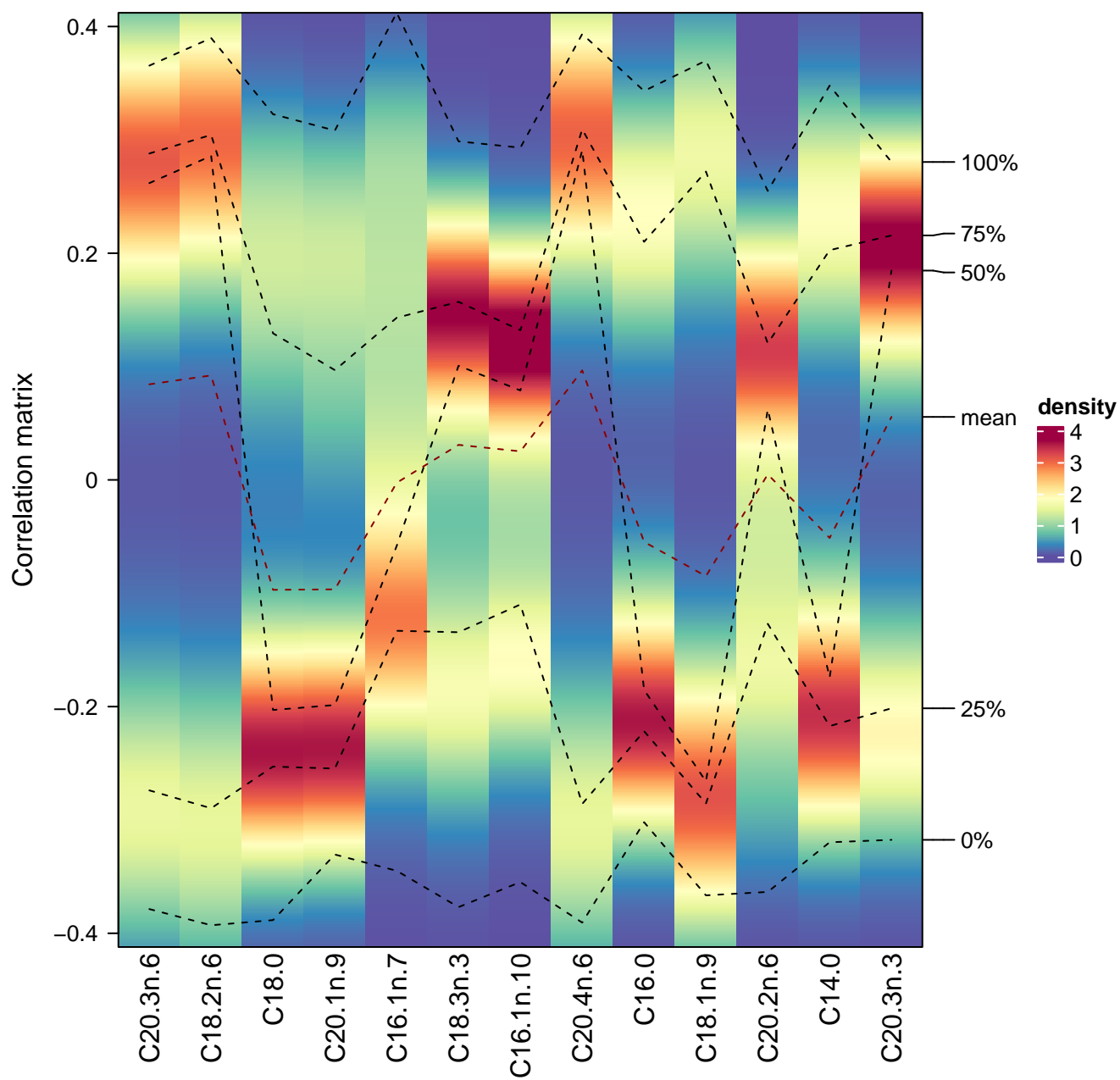

Supplement: Supplementary file 5 — Additional file 5: Figure S3. Title: Density distribution of correlation between FA and gene expression profiles, including five quantile levels and mean value in the longissimus dorsi muscle from BC1_DU pigs. Description: Distribution of correlations (FA vs. gene expression) as density heatmap using the densityHeatmap function. Here, the density was calculated by column from input data passed as a list item. [file 12711_2024_882_MOESM5_ESM.pdf]

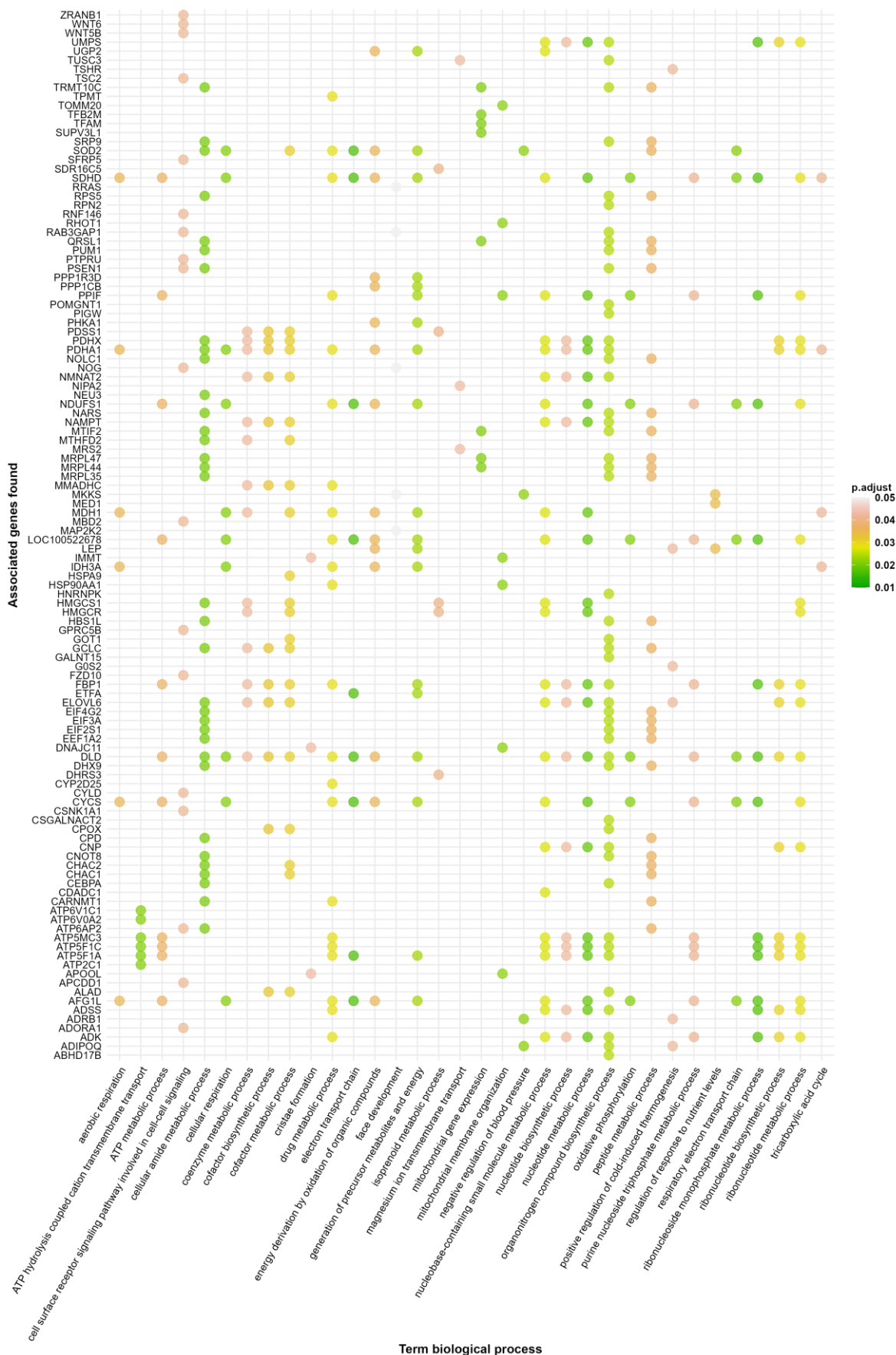

**Term biological process**

Supplement: Supplementary file 6 — Additional file 6: Figure S4. Title: Functional analysis with correlated genes from rCCA that were significantly enriched in GO terms according to delimitation of biological processes. Description: This output is a representation of the original table of results generated with the ClueGO plugin in the Cytoscape software. [file 12711_2024_882_MOESM6_ESM.pdf]
